# Human brucellosis in Portugal—Retrospective analysis of suspected clinical cases of infection from 2009 to 2016

**DOI:** 10.1371/journal.pone.0179667

**Published:** 2017-07-10

**Authors:** Ana Pelerito, Rita Cordeiro, Rita Matos, Maria Augusta Santos, Sofia Soeiro, João Santos, Carla Manita, Carla Rio, M. Santo, Eleonora Paixão, Alexandra Nunes, Sofia Núncio

**Affiliations:** 1 Emergency Response and Biopreparedness Unit, Department of Infectious Diseases, National Institute of Health, Lisbon, Portugal; 2 Immunology Laboratory, Department of Infectious Diseases, National Institute of Health, Lisbon, Portugal; 3 Immunology Laboratory, Department of Infectious Diseases, National Institute of Health, Porto, Portugal; 4 Alentejo Regional Administration of Health, Nucleus of Support in the area of Statistics, Portugal; 5 Bioinformatics Unit, Department of Infectious Diseases, National Institute of Health, Lisbon, Portugal; Universidade Federal de Minas Gerais, BRAZIL

## Abstract

Brucellosis is a zoonosis that is emerging in some regions of the world. Although brucellosis is a disease of obligatory declaration and is not eradicated in Portugal, no prevalence data is available in this country. In this study, we retrospectively analyzed the data available at the Reference Laboratory at the Portuguese National Institute of Health during the past 7 years (2009–2016) in order to get insight into the epidemiological scenario of brucellosis in Portugal. A total of 2313 biological samples from patients with clinical suspicion of brucellosis were subjected to immunological techniques for laboratory diagnosis. From 2010 to 2015, a subset of 259 samples was subjected to molecular methods. According to the available data, 167 out of 2313 (7.2%) samples had positive serology for *Brucella* spp. and 43 out of 259 samples (16.6%) were positive for *B*. *melitensis* by real time PCR, being classified as biovar 1 and 3. This study draws attention to the importance of integrating clinical and laboratory data of human cases in order to increase the efficacy of the response measures in case of outbreaks.

## Introduction

Brucellosis is a worldwide zoonosis caused by the intracellular facultative bacteria of the *Brucella* genus [1, 2]. The later currently encloses 12 species, five of which (*B*. *abortus*, *B*. *suis*, *B*. *melitensis*, *B*. *ovis* and rarely *B*. *canis*) are the ones more commonly associated with human disease [3, 4]. *B*. *melitensis* is the most virulent and has the largest public health impact in the EU due to its predominance in small ruminant populations [1]. Human brucellosis, also known as Malta fever, Undulating, Mediterranean, Gibraltar or Bang Disease, affects the well-being of people, not only as a disease in man and animals, but due to its economic impact, since it implies heavy losses in livestock farms. It also influences people’s life quality, especially those who live in rural areas, where contact with animals and the consumption of food and milk from homebred animal origin is more frequent and less controlled [5]. The two most common ways of human infection are through the contact with infected animals or the ingestion of unpasteurized dairy products. Risk groups for this disease include individuals that work with unvaccinated infected animals, farmers, slaughterhouse workers and veterinarians. They get infected through direct contact or inhalation of aerosols produced by the infected animal tissue. This situation is frequently found in areas where brucellosis is endemic in ovine and bovine cattle, and it is usually associated with infection by *B*. *melitensis* [6]. Human brucellosis is a systemic disease that may affect any organ or system, in subacute, acute or chronic form. The disease has several clinical presentations, depending on the species, the mode of transmission and also the host immune response [7]. The incubation period is difficult to determine in humans, ranging from one week to more than two months (usually 2–4 weeks)[8]. Fever, night sweats, severe headache and body aches and other non-specific symptoms may occur. Acute and chronic brucellosis can lead to complications in multiple organ systems. The musculoskeletal system, central nervous system, respiratory tract, the liver, heart, gastrointestinal and genitourinary tracts can all be affected. Untreated brucellosis has a fatality rate of 5% [9].

The inclusion of *Brucella* spp. in the list of agents with the potential to be used as a biological weapon increased the concern of the authorities responsible for human and animal health [10, 11] and made reference laboratories ensure constant improvement and update their laboratory methods for diagnosis and early detection of the pathogen in both environmental, food and biological samples [12, 13, 14]. On this regard, it is also important to have the complete information regarding phenotype and genotype of the strains that are most prevalent in each geographic region.

The laboratory diagnosis is based on the use of direct methods, such as the isolation of the causative agent for culture analysis and detection of nucleic acids by molecular methods, as well as indirect methods such as the detection of specific antibodies. However, the immunological diagnosis of human brucellosis does not differentiate the species of the genus *Brucella* spp. [12]. Recently, several molecular methods were developed, including real time PCR, which reveals great potential for direct and rapid identification of species of the genus Brucella spp. [15].

In Portugal, brucellosis is a notifiable disease, and one of the three most frequent zoonosis. Human cases are reported in all regions of continental Portugal, as shown in the 2011–2014 report of the General Directorate of Health (DGS) [16]. Nevertheless, there is no published study with data on the prevalence and incidence of human brucellosis in Portugal, so the real prevalence of brucellosis in Portugal is unknown. Moreover, for the vast majority of the reported cases it has not been possible to identify which *Brucella* species caused the infection.

This is not done and this lack of information may have serious impact in the identification of the sources of infection, impairs the identification of the most important reservoir hosts and also the implementation of timely and adequate measures that could promote the prevention and/or mitigation of the impact of this infection in the population.

The aim of this study was to contribute to a more accurate evaluation of the epidemiological situation of human brucellosis in Portugal, through the analysis of data available at the Department of Infectious Diseases at the Portuguese National Institute of Health (NIH), gathered between 2009 and 2016.

## Methods

Between 2009 and 2016, 2571 samples from patients with clinical suspicion of brucellosis were received at the Reference Laboratory at the Portuguese National Institute of Health (NIH) for diagnostic purposes. Samples were analyzed by immunological techniques, except for a subset of 259 samples (collected between 2010 and 2016) that were instead analyzed by a combination of molecular methods in agreement with the clinicians’ request. In the present study, we conducted a retrospective analysis of all data collected on those samples. No informed consent was obtained from each participant as, besides the information regarding gender and age, no further information was available to the laboratory and no tests besides the ones requested by the clinicians were performed. This procedure is in agreement with the Portuguese law No. 12/2005 of 26 January). This study was also approved by the ethical commission of National Institute of Health and the anonymity of the patients was maintained.

The immunological diagnosis of brucellosis infection was made using serological methods for antibodies’ detection based on agglutination techniques (Rose Bengal (Vircell, Granada, Spain), Wright, 2-mercaptoethanol (Fortness, Diagnostic, UK), Coombs test, indirect immunofluorescence (IFI) and immunoenzymatic assays (Brucella Elisa Igm/IgG Testkit, Virotech, Russelsheim, Germany)). All samples of sera and/or cerebrospinal fluid (CSF) were analyzed at least by two of the mentioned above immunological techniques. According to the Reference Laboratory at the Portuguese NIH guidelines, we considered a positive serological result when we observed a simultaneously positive result for one agglutination technique and one IFI or ELISA. No bacteriological cultures or PCR techniques were attempted in the serologically positive cases.

The molecular methods of brucellosis infection were performed in a tandem fashion. First, an “in house” real time PCR using hydrolysis probes was used to detect and identify the species of *Brucella* genus from blood samples, CSF, biopsies and strains isolated from blood cultures. For the rapid, sensitive and accurate detection of *Brucella* spp., the multiple IS711insertion elements were chosen as they are conserved in both number and position in the *Brucella* chromosomes [17]. For species differentiation, primers and Taqman probes were designed within the following ORFs: BMEII0466 gene for *B*. *melitensis*, BruAb2_0168 gene for *B*. *abortus* [17].

Finally to distinguish *Brucella* biovars, a molecular characterization of the *rpoB* gene was also performed. In contrast to the *16S* rRNA locus, which lacks sufficient sequence variability for differentiation of *Brucella* spp, the *rpoB* gene shows sufficient polymorphism to differentiate all *Brucella* species and their biovars; the exceptions are *B*. *abortus* biovars 1 and 4 and *B*. *abortus* biovars 5, 6 and 9, which show the same *rpoB* sequence [18].

Brucella strains were subjected to whole-genome sequencing on a MiSeq Illumina platform (Illumina) for other purposes than the ones of the present study, but allowing us to perform the *in silico* extraction of the *rpoB*. All 4134 bp *rpo*B gene sequences were retrieved from each draft genome and were compared with that of the published *B*. *melitensis* 16M genome [18]. *B*. *melitensis* strains are classified in three *rpo* types (biovar 1, biovar 2 and biovar 3) according to the presence or absence of mutations in *rpo* gene targeting the specific codon residues 629, 985, 1249 and 1309. Basically, a strain was classified as phenotypically belong to biovar 1, if *rpoB* is 100% identical to that of the *B*. *melitensis* 16M genome. The presence of nucleotide substitutions GCG to GTG at codon 629, GCC to GTC at codon position 985 and CTG to CTA at codon position 1309 underlies the classification as biovar 2. The existence of the nucleotide substitution ATG to ATA at codon position 1249 leads to the classification as biovar 3 [18].

Statistical analysis was performed using descriptive analysis and associations were tested using Chi-squared test. It was considered a 5% significance level to reject the null hypothesis of the tests. Statistical analyses were computed using software R version 3.3.2.

As this study constitutes a retrospective analysis, some of its methodological limitations regard to the lack of information that would allow a more complete analysis of risk factors (such as occupation and residence area of the patients). Also, as the analyzed samples had been sent to the lab with the clinician request for a specific diagnostic method, we respected such request hampering the use of a single method for all samples.

## Results

**Fig 1:**
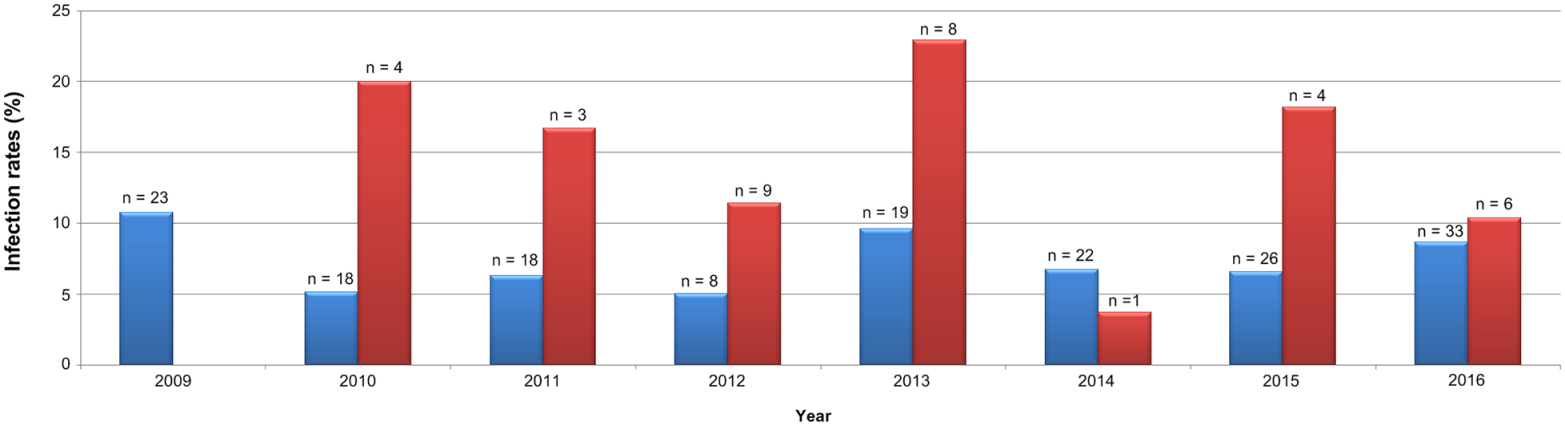
Brucellosis infection rate between 2009 and 2016. Distribution of Brucellosis cases identified in the Portuguese National Institute of Health in the period between 2009 and 2016, by immunological techniques (blue bars) and molecular biology (red bars). The “n” above each bar corresponds to the number of positive samples. Infection rate per year was defined as the number of positive cases / total number of patients. Of the 167 patients that yielded positive serology for *Brucella* spp, 61.7% (103/167) were male and 38.3% (64/167) were female (p = 0.014) (data not shown). The age was known for 98.8% (165/167) of the cases, of which half (57.7%, 95/165) are between 26–65 years. Although the distribution by age groups showed an irregular pattern, we found that 5.4% of the positive cases belong to children <5 years (Fig 2 and S1 Table).

Between January 2009 and December 2016, 2313 biological samples from patients with clinical suspicion of brucellosis were analyzed by immunological techniques and 7.2% (167/2313) had positive serology for *Brucella* spp. The distribution of infection rate by year ranged from 5% (2012) to 10.7% (2009) for the 2313 samples analyzed by immunological methods, (Fig 1 and S1 Table).

Between the years 2010 and 2016, 259 samples were tested by real time PCR methods, and 16.6% (43/259) were positive for *Brucella* spp., being *Brucella melitensis* the only species identified in the analyzed cases (Fig 1). The distribution of infection rate by year ranged from 3.7% (2014) to 22.9% (2013).

**Fig 2:**
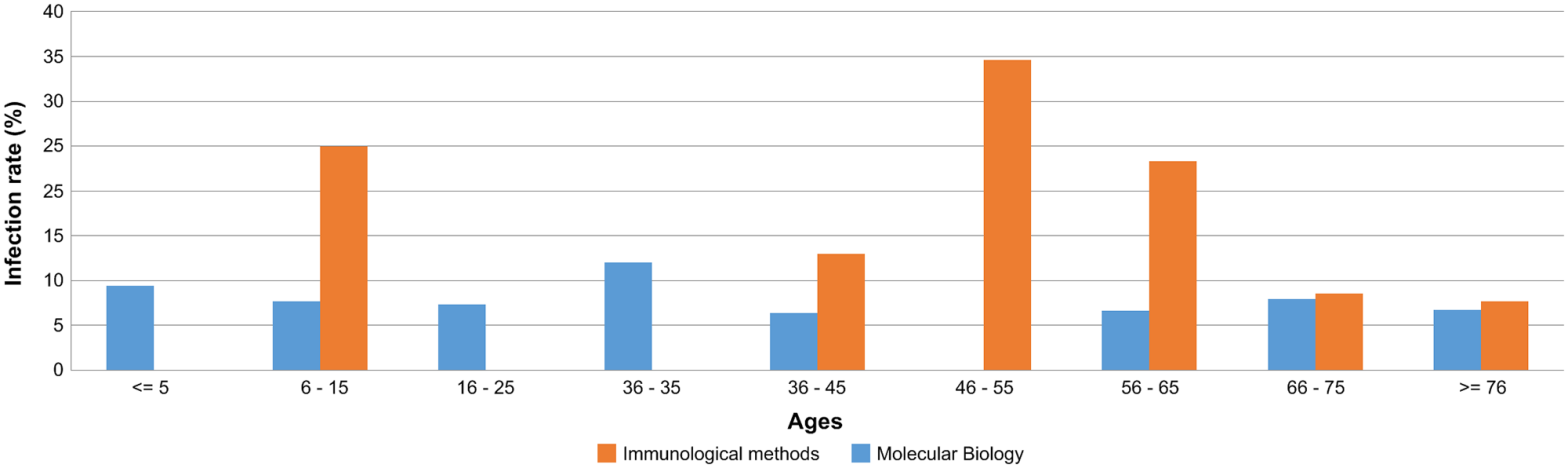
Brucellosis infection rate by age groups. Distribution of Brucellosis infection rate by age groups performed by immunological techniques (blue bars) and molecular biology (red bars).

Concerning this subset of samples, a higher prevalence of positive samples for *Brucella* spp. was observed in males (p = 0.007), similar to the scenario observed for the immunological methods. Regarding age distributions, in average, the age of the infected patients was 48.5 years (ranging between 6 and 91 years) (p<0.001) (Fig 2).

The wild-type strains were classified by analyzing the *B*. *melitensis rpo*B types. The strain frequencies for these types were 14,3% for *rpo*B biovar 1 and 85,7%, for biovar 3. None of the strains belonged to biovar 2.

## Discussion and conclusions

In the present study, we intended to shed some light on the still unveiled prevalence scenario of human brucellosis in Portugal, by conducting a retrospective study on about 2700 samples received at the Portuguese NIH over a 7-year period. Overall, serological diagnostic identified 167 (7.2%) positive cases of human brucellosis, of which 61.7% were male and half of the cases were in the age groups between 26–65 years. Preview studies show that in industrialized countries the disease mainly affects men aged between 20 and 45 years, and suggests that the distribution by gender is connected to occupational factor [19]. In fact the people who work with farm animals, especially with cattle, sheep, goats and pigs (e.g., farmers, farm laborers, animal attendants, stockmen, shepherds, sheep shearers, goatherds, pig keepers, veterinarians and inseminators) are at risk through direct contact with infected animals or through exposure to a heavily contaminated environment. Although we found an irregular pattern of distribution of brucellosis by age groups, the infection rates calculated by molecular techniques revealed that the age groups between 46 and 65 years old are among the ones with the highest rates. This falls within the range of the one described in the ECDC “Annual epidemiological report—Food-and waterborne diseases and zoonose in 2014”, in the European Union (EU), reflecting a higher number of cases registered in the age group 45 to 64 years old [20]. We also observed a low infection rate in children (5.4%), which is in agreement with data from the European Food Safety Authority, reporting that the vast majority (80%) of the European cases of brucellosis were adults over 25 years. This lower infection rate in children when compared with the one observed in adults likely relies on the low contact of children with the common infection sources, such as infected animals and animal products.

Although the molecular diagnostic was only applied to a subset of samples from 2010 to 2016, from the 259 analyzed samples, 43 (16.6%) were positive for *B*. *melitensis*. The higher infection rates obtained when using real time PCR when compared with immunological methods are likely due not only to a probable higher sensitivity of the former technique, but also because, according to our experience, PCR is usually requested when the clinician has a strong suspicion of brucellosis (e.g., patients revealing complications associated with the disease). The majority of the PCR positive cases belonged to biovar 3, pointing it as clearly the most common species/biovar involved in the human disease in Portugal. Like other countries, Portugal, applies specific regulations and measures to eradicate the disease, however, regardless of the huge efforts to eliminate it, brucellosis has continued to be an endemic disease where *B*.*melitensis* biovars 1 and 3 amd *B*. *abortus* biovars 1 and 3 are the prevailing animals species. [21]. This is in agreement with the data available for Europe. In fact, species information was provided for 99 of the 332 confirmed cases reported in the EU and Norway between 2008–2012, where 83.8% were reported to be *B*. *melitensis* [20]. Although all clinical cases in Portugal were caused by *B*. *melitensis*, other *Brucella* species pathogenic to humans have been identified in animals, namely *B*. *abortus* and *B*. *suis* [22]. This underlines the importance to perform the early detection and identification at species’ level of the *Brucella* strains obtained from clinical samples (human and animals), which is a critical information to prevent or control the occurrence of outbreaks. For this reason, molecular techniques, such as the real time PCR, particularly when applied to patients with compatible clinical symptoms and negative serological findings, are the most useful approach for laboratory diagnosis due to the rapid and precise identification of the *Brucella* sp. strain present in the clinical sample.

The geographical distribution of brucellosis is constantly changing, with the emergence and reemergence of new outbreaks around the world. Reflecting the social, cultural, and economic policies that describe a changing global society, this pathology has been reflecting this dynamics, making its control and eradication a constant challenge.

In conclusion, despite the control and prevention measures implemented by the national authorities, brucellosis remains a problem in Portugal, with impact in public health and in the economy. This study draws attention to the importance of integrating clinical and laboratory data of human cases in order to increase the efficacy of the response measures, essentially in case of outbreaks. Furthermore, our findings reinforce the need to maintain an active epidemiological surveillance, enabling the early detection of all cases of infection and underlie the need to have a good communication flow between the human and animal Health Ministries, according to the One Health concept, the only valid way to improve the assessment of the actual epidemiological situation of brucellosis and other zoonosis in Portugal.

## Supporting information

S1 Table Data of human brucellosis in Portugal. (PDF)
