# Supplementary material for: Human brucellosis in Portugal—Retrospective analysis of suspected clinical cases of infection from 2009 to 2016
Source: PLoS One. 2017 Jul 10;12(7):e0179667. doi: 10.1371/journal.pone.0179667 (PMC5503191; doi:10.1371/journal.pone.0179667)
Supplement: S1 Table — (PDF) [file pone.0179667.s001.pdf]

Imunology data

Type of Sample	Patient	Age	Sex
Soro	D1	39	M
Soro	D2	47	F
Soro	D3	53	F
Soro	D4	87	M
Soro	D5	47	M
Soro	D6	59	M
Soro	D7	54	M
Soro	D8	61	M
Soro	D9	17	F
Soro	D10	47	M
Soro	D11	69	F
Soro	D12	58	M
Soro	D13	84	F
Soro	D14	76	M
Soro	D15	66	M
Soro	D16	54	F
Soro	D17	63	M
Soro	D18	63	M
Soro	D19	60	M
Soro	D20	67	F
Soro	D21	60	F
Soro	D22	11	M
Soro	D23	11	M
Soro	D24	9	M
Soro	D25	24	F
Soro	D26	50	M
Soro	D27	7	M
Soro	D28	60	F
Soro	D29	57	M
Soro	D30	39	F
Soro	D31	36	M
Soro	D32	44	M
Soro	D33	57	M
Soro	D34	64	M
Liquor/LCR	D35	45	F
Soro	D36	26	M
Soro	D37	64	M
Soro	D38	29	M
Soro	D39	66	F
Soro	D40	66	F
Soro	D41	39	F
Soro	D42	33	M
Soro	D43	48	F
Liquor/LCR	D44	44	M
Liquor/LCR	D45	23	F
Soro	D46	44	I
Soro	D47	54	M
Soro	D48	50	M

Soro	D49	59	F
Liquor/LCR	D50	82	F
Soro	D51	50	F
Soro	D52	39	F
Soro	D53	36	M
Soro	D54	11	M
Soro	D55	35	F
Soro	D56	42	M
Soro	D57	42	F
Soro	D58	56	M
Soro	D59	69	M
Soro	D60	19	M
Soro	D61	56	F
Soro	D62	4	M
Soro	D63	13	M
Soro	D64	48	M
Soro	D65	75	M
Soro	D66	45	M
Soro	D67	61	F
Soro	D68	39	M
Soro	D69	34	M
Soro	D70	76	M
Soro	D71	76	M
Soro	D72	44	M
Soro	D73	65	M
Soro	D74	11	M
Soro	D75	0	F
Soro	D76	64	M
Soro	D77	72	F
Soro	D78	74	M
Soro	D79	67	M
Soro	D80	71	F
Soro	D81	68	F
Soro	D82	63	M
Soro	D83	42	F
Soro	D84	84	F
Soro	D85	85	M
Soro	D86	52	M
Soro	D87	68	M
Soro	D88	67	F
Soro	D89	67	F
Soro	D90	48	M
Soro	D91	74	M
Soro	D92	16	M
Soro	D93	32	M
Soro	D94	61	F
Soro	D95	60	F
Soro	D96	68	F
Soro	D97	56	M
Soro	D98	42	M

Soro	D99	45	M
Soro	D100	76	M
Soro	D101	21	M
Soro	D102	74	M
Soro	D103	73	M
Soro	D104	60	M
Soro	D105	52	F
Soro	D106	65	M
Soro	D107	65	M
Soro	D108	58	M
Soro	D109	64	M
Soro	D110	18	F
Soro	D111	72	M
Liquor/LCR	D112	58	F
Soro	D113	79	F
Soro	D114	82	F
Soro	D115	18	F
Liquor/LCR	D116	60	F
Soro	D117	78	F
Liquor/LCR	D118	72	M
Soro	D119	62	M
Soro	D120	75	M
Soro	D121	11	M
Soro	D122	76	M
Soro	D123	48	M
Soro	D124	59	F
Soro	D125	17	F
Soro	D126	53	M
Soro	D127	53	M
Soro	D128	7	I
Soro	D129	60	M
Soro	D130	73	F
Soro	D131	40	M
Soro	D132	83	M
Liquor/LCR	D133	66	M
Soro	D134	86	F
Soro	D135	83	F
Soro	D136	65	F
Soro	D137	84	F
Soro	D138	21	M
Soro	D139	46	F
Soro	D140	77	M
Soro	D141	30	M
Soro	D142	75	M
Soro	D143	56	M
Soro	D144	41	F
Soro	D145	25	M
Soro	D146	15	M
Soro	D147	28	M
Soro	D148	38	F

Soro	D149	69	F
Soro	D150	84	F
Liquor/LCR	D151	31	M
Liquor/LCR	D152	31	M
Soro	D153	75	M
Soro	D154	55	F
Soro	D155	84	M
Soro	D156	57	M
Soro	D157	22	M
Soro	D158	50	F
Soro	D159	60	M
Liquor/LCR	D160	80	F
Soro	D161	32	F
Liquor/LCR	D162	42	M
Liquor/LCR	D163	42	M
Liquor/LCR	D164	81	F
Soro	D165	80	M
Soro	D166	65	M
Soro	D167	40	F
Soro	D168	75	F
Soro	D169	23	F
Soro	D170	59	F
Soro	D171	34	F
Soro	D172	66	F
Soro	D173	64	M
Soro	D174	78	M
Soro	D175	40	F
Soro	D176	24	M
Soro	D177	17	F
Soro	D178	40	F
Liquor/LCR	D179	59	M
Soro	D180	38	F
Soro	D181	54	M
Soro	D182	44	M
Soro	D183	44	M
Soro	D184	31	F
Soro	D185	58	F
Soro	D186	62	M
Soro	D187	46	F
Soro	D188	53	M
Soro	D189	91	M
Soro	D190	71	M
Liquor/LCR	D191	0	F
Soro	D192	46	F
Soro	D193	24	F
Soro	D194	29	M
Soro	D195	42	M
Soro	D196	41	M
Soro	D197	40	F
Soro	D198	57	M

Soro	D199	41	M
Soro	D200	40	F
Soro	D201	63	M
Soro	D202	46	M
Soro	D203	46	M
Soro	D204	36	M
Liquor/LCR	D205	47	F
Soro	D206	38	F
Soro	D207	38	F
Liquor/LCR	D208	68	M
Soro	D209	60	F
Soro	D210	55	F
Soro	D211	55	F
Soro	D212	43	M
Soro	D213	82	M
Soro	D214	20	M
Soro	D215	52	F
Soro	D216	19	M
Soro	D217	20	F
Soro	D218	35	M
Soro	D219	77	M
Soro	D220	64	M
Liquor/LCR	D221	60	M
Soro	D222	9	F
Liquor/LCR	D223	39	F
Soro	D224	70	M
Soro	D225	43	F
Soro	D226	43	M
Soro	D227	67	M
Soro	D228	73	M
Soro	D229	48	F
Soro	D230	47	F
Soro	D231	40	M
Soro	D232	65	F
Soro	D233	68	F
Soro	D234	78	M
Liquor/LCR	D235	66	M
Soro	D236	41	M
Soro	D237	76	M
Soro	D238	69	M
Soro	D239	61	M
Soro	D240	36	M
Soro	D241	15	F
Soro	D242	67	F
Soro	D243	64	M
Soro	D244	69	M
Soro	D245	20	M
Soro	D246	85	M
Soro	D247	51	M
Soro	D248	58	M

Soro	D249	26	M
Soro	D250	70	M
Soro	D251	70	M
Soro	D252	79	F
Soro	D253	69	M
Soro	D254	73	M
Soro	D255	78	F
Soro	D256	49	F
Soro	D257	46	F
Soro	D258	41	F
Soro	D259	19	M
Soro	D260	68	M
Soro	D261	63	F
Soro	D262	73	F
Soro	D263	52	F
Soro	D264	36	F
Soro	D265	65	M
Soro	D266	46	M
Soro	D267	84	F
Soro	D268	74	F
Liquor/LCR	D269	58	M
Soro	D270	56	F
Soro	D271	64	M
Soro	D272	34	F
Soro	D273	70	F
Soro	D274	45	M
Soro	D275	55	F
Soro	D276	66	M
Soro	D277	48	F
Liquor/LCR	D278	22	M
Soro	D279	63	M
Soro	D280	76	F
Soro	D281	95	M
Soro	D282	59	F
Soro	D283	30	F
Soro	D284	57	M
Soro	D285	74	F
Soro	D286	74	F
Soro	D287	58	F
Soro	D288	43	F
Soro	D289	59	M
Soro	D290	67	F
Soro	D291	63	F
Soro	D292	7	M
Soro	D293	56	F
Soro	D294	42	M
Soro	D295	41	M
Soro	D296	0	M
Soro	D297	54	M
Soro	D298	47	F

Soro	D299	49	F
Soro	D300	30	F
Soro	D301	53	F
Soro	D302	68	M
Soro	D303	41	M
Soro	D304	44	F
Soro	D305	85	M
Liquor/LCR	D306	79	M
Soro	D307	63	F
Soro	D308	0	M
Soro	D309	33	F
Soro	D310	80	M
Soro	D311	43	M
Soro	D312	37	F
Soro	D313	46	M
Soro	D314	23	F
Soro	D315	65	F
Soro	D316	79	F
Soro	D317	52	F
Soro	D318	54	M
Liquor/LCR	D319	74	M
Soro	D320	37	F
Soro	D321	74	F
Soro	D322	67	F
Liquor/LCR	D323	0	F
Soro	D324	56	M
Soro	D325	41	F
Soro	D326	71	F
Soro	D327	58	F
Soro	D328	51	F
Soro	D329	59	F
Soro	D330	15	M
Soro	D331	69	M
Soro	D332	81	M
Soro	D333	49	M
Soro	D334	41	M
Soro	D335	33	M
Soro	D336	60	M
Soro	D337	14	M
Soro	D338	26	M
Soro	D339	45	M
Soro	D340	51	F
Soro	D341	21	M
Soro	D342	27	F
Soro	D343	50	M
Soro	D344	38	F
Soro	D345	79	F
Soro	D346	61	M
Soro	D347	78	F
Soro	D348	38	F

Soro	D349	77	F
Soro	D350	38	F
Soro	D351	45	F
Soro	D352	78	F
Liquor/LCR	D353	67	M
Liquor/LCR	D354	29	F
Soro	D355	31	M
Soro	D356	57	M
Soro	D357	84	F
Soro	D358	34	M
Soro	D359	48	F
Soro	D360	83	M
Soro	D361	70	F
Liquor/LCR	D362	47	M
Liquor/LCR	D363	54	M
Soro	D364	85	F
Soro	D365	60	F
Soro	D366	52	M
Soro	D367	78	F
Soro	D368	46	M
Soro	D369	61	M
Soro	D370	50	M
Soro	D371	12	M
Soro	D372	38	F
Soro	D373	60	F
Soro	D374	67	M
Soro	D375	60	M
Soro	D376	55	F
Soro	D377	66	F
Soro	D378	29	M
Soro	D379	66	M
Soro	D380	49	F
Soro	D381	39	F
Soro	D382	28	F
Soro	D383	26	M
Soro	D384	67	F
Soro	D385	81	M
Soro	D386	69	M
Soro	D387	50	F
Soro	D388	58	M
Soro	D389	48	M
Soro	D390	53	F
Soro	D391	85	F
Soro	D392	46	F
Soro	D393	49	F
Soro	D394	28	M
Soro	D395	56	F
Soro	D396	59	F
Soro	D397	26	F
Liquor/LCR	D398	33	M

Soro	D399	18	M
Soro	D400	38	F
Soro	D401	10	F
Soro	D402	12	F
Soro	D403	54	M
Soro	D404	72	M
Soro	D405	77	M
Soro	D406	53	M
Liquor/LCR	D407	79	M
Soro	D408	44	F
Soro	D409	57	M
Soro	D410	25	F
Soro	D411	55	F
Soro	D412	43	F
Soro	D413	34	F
Soro	D414	54	M
Soro	D415	61	F
Soro	D416	83	F
Soro	D417	63	F
Liquor/LCR	D418	0	M
Soro	D419	54	M
Soro	D420	27	F
Soro	D421	26	F
Soro	D422	26	F
Soro	D423	42	F
Soro	D424	51	F
Soro	D425	71	M
Soro	D426	58	M
Liquor/LCR	D427	41	M
Soro	D428	32	M
Soro	D429	49	M
Soro	D430	61	M
Soro	D431	61	M
Soro	D432	61	F
Soro	D433	56	F
Soro	D434	47	M
Soro	D435	83	F
Soro	D436	3	F
Liquor/LCR	D437	81	M
Soro	D438	40	M
Liquor/LCR	D439	47	F
Soro	D440	38	I
Soro	D441	61	M
Soro	D442	44	F
Soro	D443	79	F
Soro	D444	56	M
Soro	D445	35	M
Soro	D446	58	M
Soro	D447	42	F
Soro	D448	63	F

Soro	D449	40	M
Soro	D450	73	F
Soro	D451	48	M
Soro	D452	14	M
Soro	D453	55	M
Soro	D454	3	F
Liquor/LCR	D455	49	M
Soro	D456	13	M
Soro	D457	13	M
Soro	D458	54	M
Soro	D459	50	F
Soro	D460	60	M
Soro	D461	55	M
Soro	D462	64	F
Liquor/LCR	D463	65	F
Soro	D464	42	M
Soro	D465	62	F
Soro	D466	25	M
Soro	D467	65	M
Soro	D468	41	M
Soro	D469	12	M
Soro	D470	54	M
Soro	D471	61	M
Soro	D472	68	F
Soro	D473	41	M
Soro	D474	79	M
Soro	D475	48	M
Soro	D476	56	M
Liquor/LCR	D477	65	M
Soro	D478	79	F
Soro	D479	58	M
Soro	D480	33	M
Soro	D481	34	M
Soro	D482	31	F
Soro	D483	85	M
Liquor/LCR	D484	79	M
Soro	D485	15	F
Soro	D486	38	M
Soro	D487	65	M
Soro	D488	69	M
Soro	D489	78	M
Soro	D490	69	F
Soro	D491	16	M
Soro	D492	76	F
Soro	D493	83	M
Soro	D494	68	M
Soro	D495	70	M
Soro	D496	32	F
Soro	D497	38	F
Soro	D498	28	M

Soro	D499	24	M
Liquor/LCR	D500	47	M
Soro	D501	62	F
Liquor/LCR	D502	71	F
Soro	D503	34	M
Soro	D504	34	M
Soro	D505	50	F
Soro	D506	34	F
Liquor/LCR	D507	88	F
Soro	D508	44	M
Soro	D509	44	F
Soro	D510	42	M
Soro	D511	38	M
Liquor/LCR	D512	40	M
Soro	D513	70	F
Liquor/LCR	D514	61	M
Soro	D515	50	F
Soro	D516	75	M
Soro	D517	79	M
Soro	D518	57	M
Soro	D519	61	M
Soro	D520	47	F
Soro	D521	39	F
Soro	D522	38	F
Soro	D523	75	F
Liquor/LCR	D524	61	M
Liquor/LCR	D525	42	M
Soro	D526	73	M
Soro	D527	61	M
Liquor/LCR	D528	57	M
Soro	D529	90	F
Liquor/LCR	D530	0	M
Soro	D531	73	F
Soro	D532	66	M
Soro	D533	69	M
Soro	D534	63	M
Soro	D535	67	F
Soro	D536	40	F
Soro	D537	76	F
Soro	D538	76	F
Soro	D539	61	M
Soro	D540	65	M
Soro	D541	63	F
Soro	D542	40	F
Soro	D543	76	F
Soro	D544	42	M
Soro	D545	60	M
Soro	D546	56	M
Soro	D547	56	F
Soro	D548	80	F

Soro	D549	75	F
Liquor/LCR	D550	82	F
Soro	D551	72	M
Soro	D552	27	M
Liquor/LCR	D553	46	F
Soro	D554	83	M
Soro	D555	52	M
Soro	D556	68	M
Soro	D557	77	F
Liquor/LCR	D558	54	F
Soro	D559	56	M
Soro	D560	63	F
Soro	D561	81	F
Soro	D562	66	M
Soro	D563	37	M
Soro	D564	50	M
Soro	D565	54	M
Soro	D566	39	M
Soro	D567	66	M
Soro	D568	71	F
Soro	D569	45	I
Soro	D570	53	M
Soro	D571	21	F
Soro	D572	33	F
Soro	D573	58	F
Soro	D574	43	F
Soro	D575	43	F
Soro	D576	44	F
Soro	D577	22	M
Soro	D578	76	F
Soro	D579	57	F
Soro	D580	63	F
Soro	D581	65	M
Soro	D582	43	M
Soro	D583	46	F
Soro	D584	58	M
Soro	D585	48	M
Soro	D586	65	M
Soro	D587	63	F
Liquor/LCR	D588	34	M
Soro	D589	51	M
Soro	D590	38	F
Soro	D591	5	M
Soro	D592	61	F
Soro	D593	45	F
Liquor/LCR	D594	59	F
Soro	D595	42	M
Soro	D596	55	M
Soro	D597	82	M
Soro	D598	84	F

Soro	D599	61	M
Soro	D600	8	F
Soro	D601	85	F
Soro	D602	73	F
Soro	D603	78	F
Soro	D604	81	M
Soro	D605	40	F
Soro	D606	70	M
Soro	D607	57	M
Soro	D608	57	M
Soro	D609	49	F
Soro	D610	42	M
Soro	D611	36	M
Soro	D612	24	F
Soro	D613	80	F
Soro	D614	61	M
Soro	D615	62	F
Soro	D616	79	M
Soro	D617	26	F
Soro	D618	84	M
Soro	D619	59	F
Soro	D620	24	F
Soro	D621	62	F
Soro	D622	49	F
Soro	D623	8	M
Soro	D624	42	F
Soro	D625	41	F
Soro	D626	36	M
Soro	D627	68	M
Soro	D628	46	F
Soro	D629	68	M
Liquor/LCR	D630	59	M
Liquor/LCR	D631	0	M
Soro	D632	76	M
Soro	D633	68	F
Soro	D634	38	M
Soro	D635	12	M
Soro	D636	54	M
Soro	D637	66	F
Liquor/LCR	D638	35	F
Soro	D639	50	F
Soro	D640	58	M
Soro	D641	48	M
Soro	D642	53	M
Soro	D643	73	M
Soro	D644	20	F
Soro	D645	63	M
Soro	D646	45	M
Soro	D647	53	F
Soro	D648	46	F

Soro	D649	14	F
Soro	D650	21	M
Soro	D651	87	F
Soro	D652	28	F
Soro	D653	72	F
Soro	D654	42	M
Soro	D655	45	M
Soro	D656	45	F
Soro	D657	44	F
Soro	D658	44	F
Soro	D659	72	M
Soro	D660	21	M
Soro	D661	61	M
Soro	D662	81	M
Soro	D663	71	M
Soro	D664	73	F
Soro	D665	27	F
Soro	D666	27	M
Soro	D667	46	F
Soro	D668	76	M
Soro	D669	3	M
Soro	D670	81	F
Soro	D671	64	M
Soro	D672	56	F
Soro	D673	58	F
Soro	D674	41	F
Soro	D675	81	F
Soro	D676	36	M
Soro	D677	57	M
Soro	D678	7	M
Soro	D679	44	M
Soro	D680	77	M
Soro	D681	56	M
Soro	D682	52	F
Soro	D683	74	M
Soro	D684	52	M
Soro	D685	38	F
Soro	D686	81	F
Soro	D687	52	M
Soro	D688	72	M
Soro	D689	60	F
Soro	D690	41	F
Soro	D691	76	M
Soro	D692	24	F
Soro	D693	33	M
Soro	D694	41	M
Soro	D695	31	F
Soro	D696	57	F
Soro	D697	58	M
Soro	D698	72	M

Soro	D699	18	M
Soro	D700	41	M
Soro	D701	57	F
Liquor/LCR	D702	24	F
Soro	D703	51	F
Soro	D704	64	M
Soro	D705	71	M
Soro	D706	59	M
Soro	D707	67	F
Soro	D708	54	M
Soro	D709	54	F
Soro	D710	64	F
Soro	D711	52	F
Soro	D712	37	F
Soro	D713	23	F
Soro	D714	64	F
Soro	D715	30	F
Soro	D716	63	F
Soro	D717	59	M
Liquor/LCR	D718	40	F
Soro	D719	36	M
Soro	D720	61	M
Soro	D721	37	M
Soro	D722	42	M
Soro	D723	46	M
Soro	D724	77	M
Soro	D725	68	M
Soro	D726	80	M
Soro	D727	29	F
Soro	D728	57	M
Soro	D729	56	M
Soro	D730	36	F
Soro	D731	26	F
Soro	D732	84	M
Soro	D733	72	F
Soro	D734	52	M
Soro	D735	57	M
Soro	D736	36	M
Soro	D737	53	M
Soro	D738	53	M
Soro	D739	81	M
Soro	D740	63	M
Soro	D741	43	M
Soro	D742	71	F
Soro	D743	6	F
Soro	D744	7	F
Soro	D745	69	M
Soro	D746	6	M
Soro	D747	86	F
Soro	D748	82	F

Soro	D749	15	F
Soro	D750	32	F
Soro	D751	68	F
Soro	D752	49	M
Liquor/LCR	D753	77	F
Soro	D754	40	M
Soro	D755	45	F
Soro	D756	81	F
Soro	D757	58	M
Soro	D758	44	M
Soro	D759	5	F
Soro	D760	5	M
Soro	D761	4	M
Soro	D762	62	M
Soro	D763	51	M
Soro	D764	34	F
Soro	D765	21	F
Soro	D766	49	F
Soro	D767	45	F
Liquor/LCR	D768	67	F
Soro	D769	67	M
Soro	D770	15	F
Soro	D771	77	M
Soro	D772	18	F
Soro	D773	61	M
Soro	D774	51	M
Soro	D775	58	F
Soro	D776	49	F
Soro	D777	43	M
Soro	D778	36	M
Soro	D779	15	M
Soro	D780	77	M
Soro	D781	69	M
Soro	D782	66	F
Soro	D783	58	F
Soro	D784	55	M
Soro	D785	37	F
Soro	D786	45	I
Soro	D787	61	M
Soro	D788	63	M
Soro	D789	81	F
Soro	D790	56	M
Soro	D791	63	F
Soro	D792	30	F
Soro	D793	46	F
Soro	D794	23	F
Soro	D795	5	M
Soro	D796	58	M
Soro	D797	85	M
Soro	D798	42	F

Soro	D799	52	M
Soro	D800	9	F
Soro	D801	19	M
Soro	D802	80	M
Soro	D803	42	F
Soro	D804	36	M
Soro	D805	50	F
Soro	D806	57	F
Soro	D807	60	F
Soro	D808	5	M
Soro	D809	33	F
Soro	D810	52	M
Soro	D811	78	M
Soro	D812	45	M
Soro	D813	58	F
Soro	D814	74	F
Soro	D815	45	M
Soro	D816	34	M
Soro	D817	57	F
Soro	D818	43	F
Soro	D819	44	M
Soro	D820	72	F
Soro	D821	67	F
Soro	D822	54	M
Soro	D823	11	M
Liquor/LCR	D824	27	M
Soro	D825	25	F
Soro	D826	42	F
Soro	D827	13	F
Soro	D828	89	F
Liquor/LCR	D829	37	F
Soro	D830	80	M
Soro	D831	48	F
Soro	D832	23	F
Soro	D833	47	F
Soro	D834	62	F
Soro	D835	54	F
Soro	D836	47	F
Soro	D837	71	F
Soro	D838	69	M
Soro	D839	52	M
Soro	D840	59	M
Soro	D841	61	F
Soro	D842	20	F
Soro	D843	41	M
Soro	D844	48	M
Soro	D845	58	F
Soro	D846	52	M
Soro	D847	11	M
Soro	D848	52	M

Soro	D849	59	F
Soro	D850	86	M
Soro	D851	72	I
Soro	D852	83	F
Liquor/LCR	D853	78	F
Soro	D854	47	F
Soro	D855	41	F
Soro	D856	51	M
Soro	D857	36	M
Soro	D858	58	M
Soro	D859	52	M
Soro	D860	49	M
Soro	D861	32	F
Soro	D862	80	M
Soro	D863	37	M
Soro	D864	43	F
Soro	D865	55	F
Soro	D866	63	F
Soro	D867	49	M
Soro	D868	55	M
Soro	D869	45	F
Soro	D870	45	F
Soro	D871	79	M
Soro	D872	36	M
Soro	D873	60	F
Soro	D874	42	F
Liquor/LCR	D875	57	M
Soro	D876	54	M
Soro	D877	64	M
Soro	D878	41	F
Soro	D879	50	M
Soro	D880	62	M
Soro	D881	42	F
Soro	D882	44	F
Soro	D883	28	F
Soro	D884	57	F
Soro	D885	35	F
Soro	D886	57	F
Soro	D887	3	M
Liquor/LCR	D888	35	F
Soro	D889	84	F
Soro	D890	35	M
Soro	D891	35	M
Soro	D892	54	F
Soro	D893	77	F
Soro	D894	59	F
Soro	D895	85	M
Liquor/LCR	D896	25	M
Soro	D897	41	F
Soro	D898	79	F

Soro	D899	35	F
Soro	D900	72	M
Soro	D901	53	F
Soro	D902	59	M
Soro	D903	36	M
Soro	D904	65	M
Soro	D905	49	F
Soro	D906	53	F
Soro	D907	56	F
Soro	D908	60	F
Soro	D909	53	F
Soro	D910	64	M
Soro	D911	47	F
Soro	D912	44	M
Soro	D913	83	F
Soro	D914	32	F
Soro	D915	57	F
Soro	D916	38	M
Soro	D917	43	M
Soro	D918	55	F
Soro	D919	17	M
Soro	D920	82	F
Soro	D921	70	M
Soro	D922	30	M
Soro	D923	44	F
Soro	D924	71	M
Soro	D925	65	F
Soro	D926	65	F
Soro	D927	30	F
Soro	D928	64	M
Soro	D929	71	F
Soro	D930	39	F
Soro	D931	80	F
Soro	D932	25	F
Soro	D933	55	M
Soro	D934	48	F
Soro	D935	56	F
Liquor/LCR	D936	46	F
Soro	D937	19	M
Soro	D938	81	F
Soro	D939	72	F
Soro	D940	22	F
Soro	D941	58	F
Soro	D942	57	M
Soro	D943	49	M
Soro	D944	36	M
Soro	D945	50	F
Soro	D946	36	M
Soro	D947	82	F
Soro	D948	70	M

Soro	D949	21	M
Soro	D950	56	M
Soro	D951	44	M
Soro	D952	14	M
Soro	D953	68	F
Soro	D954	59	F
Soro	D955	77	F
Soro	D956	83	M
Soro	D957	20	F
Soro	D958	63	F
Soro	D959	39	M
Soro	D960	42	F
Liquor/LCR	D961	46	F
Soro	D962	18	M
Soro	D963	3	F
Soro	D964	34	F
Soro	D965	52	F
Soro	D966	45	F
Soro	D967	43	M
Soro	D968	57	F
Soro	D969	71	M
Soro	D970	71	M
Soro	D971	42	M
Soro	D972	14	F
Soro	D973	39	F
Soro	D974	39	F
Soro	D975	52	F
Liquor/LCR	D976	72	F
Soro	D977	20	F
Soro	D978	16	M
Soro	D979	43	F
Soro	D980	46	F
Soro	D981	29	F
Soro	D982	63	M
Soro	D983	32	M
Soro	D984	48	F
Soro	D985	76	F
Soro	D986	45	M
Soro	D987	25	F
Soro	D988	54	F
Soro	D989	56	M
Soro	D990	50	F
Soro	D991	43	M
Soro	D992	45	M
Soro	D993	17	F
Soro	D994	62	F
Soro	D995	36	M
Soro	D996	20	F
Liquor/LCR	D997	40	M
Liquor/LCR	D998	40	M

Soro	D999	59	F
Soro	D1000	57	M
Soro	D1001	58	F
Soro	D1002	84	M
Soro	D1003	62	M
Soro	D1004	53	F
Soro	D1005	21	M
Soro	D1006	81	F
Soro	D1007	24	M
Soro	D1008	69	M
Liquor/LCR	D1009	81	F
Soro	D1010	53	M
Soro	D1011	43	M
Soro	D1012	27	M
Soro	D1013	68	M
Soro	D1014	26	M
Soro	D1015	64	M
Soro	D1016	60	M
Liquor/LCR	D1017	74	M
Soro	D1018	20	F
Soro	D1019	29	F
Soro	D1020	19	M
Soro	D1021	51	F
Soro	D1022	59	M
Soro	D1023	62	F
Soro	D1024	22	F
Soro	D1025	66	M
Soro	D1026	67	F
Soro	D1027	40	F
Soro	D1028	44	M
Soro	D1029	57	M
Soro	D1030	64	M
Soro	D1031	24	M
Soro	D1032	7	M
Soro	D1033	55	M
Soro	D1034	38	M
Soro	D1035	82	M
Soro	D1036	79	M
Soro	D1037	16	F
Soro	D1038	57	M
Soro	D1039	78	M
Soro	D1040	16	F
Soro	D1041	34	M
Soro	D1042	45	F
Soro	D1043	50	M
Soro	D1044	50	M
Soro	D1045	22	F
Soro	D1046	62	M
Soro	D1047	23	F
Soro	D1048	33	M

Soro	D1049	65	M
Soro	D1050	57	M
Liquor/LCR	D1051	61	M
Soro	D1052	26	M
Soro	D1053	62	F
Soro	D1054	55	F
Soro	D1055	20	M
Soro	D1056	24	F
Soro	D1057	60	M
Soro	D1058	46	F
Soro	D1059	58	M
Soro	D1060	45	F
Soro	D1061	30	F
Soro	D1062	52	M
Soro	D1063	75	F
Soro	D1064	5	M
Soro	D1065	77	F
Soro	D1066	6	F
Soro	D1067	63	M
Soro	D1068	56	M
Soro	D1069	13	M
Soro	D1070	27	F
Soro	D1071	49	M
Soro	D1072	66	F
Soro	D1073	46	M
Soro	D1074	14	M
Soro	D1075	44	M
Soro	D1076	55	M
Soro	D1077	47	M
Soro	D1078	18	M
Soro	D1079	72	F
Soro	D1080	13	M
Soro	D1081	49	F
Soro	D1082	12	M
Soro	D1083	60	M
Liquor/LCR	D1084	45	F
Soro	D1085	47	F
Soro	D1086	57	F
Soro	D1087	78	M
Soro	D1088	65	M
Soro	D1089	70	F
Soro	D1090	55	M
Soro	D1091	47	M
Soro	D1092	13	F
Soro	D1093	49	M
Soro	D1094	50	F
Soro	D1095	29	M
Soro	D1096	79	F
Soro	D1097	19	F
Soro	D1098	73	F

Soro	D1099	70	M
Soro	D1100	49	M
Soro	D1101	71	M
Soro	D1102	67	M
Soro	D1103	54	M
Soro	D1104	28	F
Soro	D1105	40	M
Soro	D1106	53	M
Soro	D1107	27	F
Soro	D1108	66	M
Soro	D1109	43	F
Soro	D1110	33	M
Soro	D1111	35	M
Soro	D1112	36	F
Soro	D1113	75	M
Soro	D1114	38	M
Soro	D1115	56	F
Soro	D1116	38	M
Soro	D1117	34	F
Soro	D1118	64	F
Soro	D1119	72	F
Soro	D1120	53	M
Soro	D1121	68	F
Soro	D1122	48	M
Soro	D1123	65	M
Soro	D1124	52	F
Soro	D1125	63	M
Soro	D1126	23	F
Soro	D1127	55	M
Soro	D1128	42	F
Soro	D1129	62	F
Soro	D1130	64	F
Soro	D1131	57	M
Soro	D1132	24	F
Soro	D1133	54	F
Liquor/LCR	D1134	32	M
Soro	D1135	54	M
Soro	D1136	59	M
Soro	D1137	65	M
Soro	D1138	58	F
Soro	D1139	33	F
Soro	D1140	21	F
Soro	D1141	68	M
Soro	D1142	53	M
Soro	D1143	17	M
Soro	D1144	34	F
Soro	D1145	41	M
Soro	D1146	33	M
Soro	D1147	54	F
Soro	D1148	85	M

Soro	D1149	56	M
Soro	D1150	42	M
Soro	D1151	35	F
Soro	D1152	49	M
Soro	D1153	50	F
Soro	D1154	24	M
Soro	D1155	53	M
Soro	D1156	51	F
Soro	D1157	44	M
Soro	D1158	76	M
Soro	D1159	38	F
Soro	D1160	39	F
Soro	D1161	24	M
Soro	D1162	56	M
Soro	D1163	36	F
Soro	D1164	6	F
Soro	D1165	61	M
Soro	D1166	63	M
Soro	D1167	89	F
Soro	D1168	36	M
Soro	D1169	57	M
Soro	D1170	52	F
Soro	D1171	23	M
Soro	D1172	60	M
Soro	D1173	49	M
Soro	D1174	66	M
Soro	D1175	52	F
Soro	D1176	59	M
Soro	D1177	3	M
Soro	D1178	15	M
Soro	D1179	39	F
Soro	D1180	21	F
Soro	D1181	31	M
Soro	D1182	51	M
Soro	D1183	29	F
Soro	D1184	66	M
Soro	D1185	75	M
Soro	D1186	63	F
Soro	D1187	63	F
Soro	D1188	50	F
Soro	D1189	23	F
Soro	D1190	55	M
Soro	D1191	80	M
Soro	D1192	60	M
Soro	D1193	52	M
Soro	D1194	45	M
Soro	D1195	49	F
Soro	D1196	49	F
Soro	D1197	66	F
Soro	D1198	20	M

Soro	D1199	51	M
Soro	D1200	55	M
Soro	D1201	77	F
Soro	D1202	67	F
Soro	D1203	65	M
Soro	D1204	54	F
Soro	D1205	58	M
Soro	D1206	89	M
Soro	D1207	24	F
Soro	D1208	50	F
Soro	D1209	62	M
Soro	D1210	8	M
Soro	D1211	11	M
Soro	D1212	36	M
Soro	D1213	37	F
Soro	D1214	39	F
Liquor/LCR	D1215	65	F
Soro	D1216	83	F
Soro	D1217	59	F
Soro	D1218	63	M
Soro	D1219	63	M
Soro	D1220	42	F
Soro	D1221	82	M
Soro	D1222	61	M
Soro	D1223	84	M
Soro	D1224	55	M
Soro	D1225	34	F
Soro	D1226	52	M
Soro	D1227	63	F
Soro	D1228	42	M
Soro	D1229	31	M
Soro	D1230	83	F
Soro	D1231	61	M
Soro	D1232	75	F
Soro	D1233	62	M
Soro	D1234	16	F
Soro	D1235	83	F
Soro	D1236	65	F
Liquor/LCR	D1237	82	F
Soro	D1238	57	M
Soro	D1239	86	F
Soro	D1240	39	M
Soro	D1241	52	F
Soro	D1242	63	M
Soro	D1243	72	F
Soro	D1244	44	M
Soro	D1245	70	M
Soro	D1246	56	F
Liquor/LCR	D1247	47	M
Liquor/LCR	D1248	53	M

Soro	D1249	59	F
Soro	D1250	52	M
Soro	D1251	42	M
Soro	D1252	65	M
Soro	D1253	49	M
Soro	D1254	38	F
Soro	D1255	87	F
Soro	D1256	37	M
Soro	D1257	55	F
Soro	D1258	73	F
Soro	D1259	43	M
Soro	D1260	61	M
Soro	D1261	48	F
Soro	D1262	60	F
Soro	D1263	78	F
Soro	D1264	78	F
Soro	D1265	54	M
Soro	D1266	66	M
Soro	D1267	58	F
Soro	D1268	74	M
Soro	D1269	41	M
Liquor/LCR	D1270	30	F
Soro	D1271	33	F
Soro	D1272	38	F
Soro	D1273	40	F
Soro	D1274	39	M
Liquor/LCR	D1275	75	M
Soro	D1276	64	M
Liquor/LCR	D1277	33	M
Liquor/LCR	D1278	45	M
Liquor/LCR	D1279	41	F
Soro	D1280	57	M
Soro	D1281	26	M
Soro	D1282	61	F
Soro	D1283	49	F
Soro	D1284	67	F
Soro	D1285	49	F
Soro	D1286	64	F
Liquor/LCR	D1287	42	M
Liquor/LCR	D1288	50	M
Liquor/LCR	D1289	50	M
Liquor/LCR	D1290	74	F
Soro	D1291	54	M
Soro	D1292	68	F
Liquor/LCR	D1293	44	F
Soro	D1294	86	M
Soro	D1295	53	M
Soro	D1296	52	F
Soro	D1297	81	M
Soro	D1298	15	M

Soro	D1299	20	F
Soro	D1300	75	F
Soro	D1301	25	M
Soro	D1302	79	F
Soro	D1303	60	F
Soro	D1304	55	M
Soro	D1305	31	M
Soro	D1306	33	F
Soro	D1307	39	F
Soro	D1308	7	M
Soro	D1309	82	F
Soro	D1310	70	F
Soro	D1311	34	M
Soro	D1312	81	M
Soro	D1313	30	M
Soro	D1314	39	M
Soro	D1315	62	F
Soro	D1316	65	F
Soro	D1317	42	M
Soro	D1318	35	M
Soro	D1319	70	F
Soro	D1320	69	M
Soro	D1321	59	F
Soro	D1322	64	M
Soro	D1323	83	M
Soro	D1324	33	F
Liquor/LCR	D1325	84	F
Liquor/LCR	D1326	84	M
Soro	D1327	25	M
Soro	D1328	71	F
Soro	D1329	38	F
Soro	D1330	74	M
Soro	D1331	66	M
Soro	D1332	78	M
Liquor/LCR	D1333	84	M
Soro	D1334	81	F
Soro	D1335	71	M
Soro	D1336	29	M
Liquor/LCR	D1337	41	M
Soro	D1338	42	F
Soro	D1339	71	F
Soro	D1340	84	M
Soro	D1341	78	F
Soro	D1342	78	M
Soro	D1343	71	M
Liquor/LCR	D1344	70	M
Soro	D1345	75	F
Soro	D1346	72	F
Soro	D1347	23	F
Soro	D1348	73	M

Soro	D1349	44	F
Soro	D1350	24	F
Soro	D1351	35	M
Soro	D1352	44	F
Soro	D1353	44	F
Soro	D1354	92	M
Soro	D1355	55	F
Soro	D1356	86	F
Soro	D1357	44	M
Soro	D1358	37	F
Soro	D1359	67	M
Soro	D1360	44	F
Soro	D1361	49	F
Soro	D1362	69	F
Soro	D1363	33	I
Liquor/LCR	D1364	31	F
Soro	D1365	51	M
Soro	D1366	38	F
Soro	D1367	74	F
Soro	D1368	82	F
Soro	D1369	66	M
Soro	D1370	80	M
Soro	D1371	61	M
Soro	D1372	47	M
Soro	D1373	55	F
Soro	D1374	55	M
Soro	D1375	32	M
Soro	D1376	73	M
Soro	D1377	74	M
Liquor/LCR	D1378	50	M
Soro	D1379	53	M
Soro	D1380	66	M
Soro	D1381	87	F
Soro	D1382	91	F
Soro	D1383	37	I
Soro	D1384	90	F
Soro	D1385	80	F
Soro	D1386	85	M
Soro	D1387	54	F
Soro	D1388	25	M
Soro	D1389	83	M
Soro	D1390	83	M
Soro	D1391	11	F
Soro	D1392	52	F
Soro	D1393	90	M
Soro	D1394	49	F
Soro	D1395	28	M
Soro	D1396	71	M
Soro	D1397	72	F
Soro	D1398	77	M

Liquor/LCR	D1399	52	F
Soro	D1400	35	F
Soro	D1401	47	M
Soro	D1402	55	M
Liquor/LCR	D1403	56	M
Soro	D1404	54	F
Soro	D1405	19	F
Soro	D1406	51	M
Soro	D1407	24	F
Soro	D1408	54	F
Soro	D1409	60	F
Soro	D1410	51	F
Soro	D1411	37	F
Soro	D1412	79	F
Soro	D1413	44	M
Soro	D1414	64	F
Soro	D1415	72	F
Soro	D1416	67	F
Soro	D1417	70	M
Soro	D1418	60	F
Soro	D1419	42	F
Soro	D1420	39	F
Soro	D1421	71	M
Soro	D1422	73	M
Soro	D1423	40	M
Soro	D1424	28	F
Soro	D1425	42	F
Soro	D1426	43	M
Soro	D1427	69	F
Soro	D1428	49	M
Liquor/LCR	D1429	49	F
Soro	D1430	33	F
Liquor/LCR	D1431	85	F
Soro	D1432	88	F
Soro	D1433	69	F
Soro	D1434	55	F
Soro	D1435	76	M
Soro	D1436	59	F
Liquor/LCR	D1437	40	M
Soro	D1438	66	M
Soro	D1439	84	M
Soro	D1440	75	M
Soro	D1441	33	F
Soro	D1442	45	F
Liquor/LCR	D1443	40	F
Soro	D1444	67	M
Soro	D1445	35	M
Soro	D1446	51	M
Soro	D1447	66	F
Soro	D1448	28	F

Soro	D1449	63	F
Soro	D1450	61	M
Soro	D1451	55	F
Soro	D1452	48	F
Soro	D1453	35	F
Soro	D1454	0	M
Soro	D1455	0	M
Soro	D1456	40	M
Soro	D1457	47	M
Liquor/LCR	D1458	92	M
Soro	D1459	11	F
Liquor/LCR	D1460	87	M
Soro	D1461	83	F
Liquor/LCR	D1462	30	F
Soro	D1463	46	M
Soro	D1464	80	F
Soro	D1465	54	F
Soro	D1466	29	M
Soro	D1467	25	M
Soro	D1468	25	M
Soro	D1469	48	F
Soro	D1470	11	M
Soro	D1471	82	F
Soro	D1472	32	F
Soro	D1473	56	M
Soro	D1474	85	F
Soro	D1475	39	M
Soro	D1476	85	M
Soro	D1477	52	M
Soro	D1478	50	F
Soro	D1479	47	F
Soro	D1480	36	F
Soro	D1481	59	F
Soro	D1482	31	F
Liquor/LCR	D1483	51	M
Liquor/LCR	D1484	53	F
Soro	D1485	62	F
Soro	D1486	53	F
Soro	D1487	36	M
Soro	D1488	51	F
Soro	D1489	62	F
Soro	D1490	63	F
Soro	D1491	78	F
Soro	D1492	0	F
Soro	D1493	77	F
Soro	D1494	35	F
Liquor/LCR	D1495	63	F
Soro	D1496	69	F
Soro	D1497	49	M
Soro	D1498	66	F

Soro	D1499	49	F
Soro	D1500	85	M
Soro	D1501	0	M
Soro	D1502	36	F
Soro	D1503	63	F
Soro	D1504	71	F
Soro	D1505	75	F
Soro	D1506	68	M
Soro	D1507	47	F
Soro	D1508	81	M
Soro	D1509	37	F
Soro	D1510	0	F
Soro	D1511	91	M
Soro	D1512	36	M
Soro	D1513	85	M
Soro	D1514	49	F
Soro	D1515	45	F
Soro	D1516	65	F
Soro	D1517	61	M
Soro	D1518	36	F
Soro	D1519	35	M
Soro	D1520	29	M
Soro	D1521	66	F
Soro	D1522	51	F
Soro	D1523	72	F
Soro	D1524	32	F
Soro	D1525	33	F
Soro	D1526	62	F
Soro	D1527	0	M
Liquor/LCR	D1528	63	M
Soro	D1529	48	M
Soro	D1530	74	M
Soro	D1531	45	F
Soro	D1532	48	M
Liquor/LCR	D1533	55	M
Soro	D1534	12	F
Soro	D1535	75	F
Soro	D1536	56	M
Soro	D1537	70	F
Soro	D1538	34	F
Soro	D1539	38	M
Soro	D1540	76	M
Soro	D1541	20	F
Soro	D1542	83	F
Soro	D1543	72	F
Soro	D1544	24	M
Soro	D1545	63	F
Soro	D1546	34	F
Soro	D1547	83	F
Soro	D1548	45	F

Soro	D1549	79	M
Soro	D1550	0	M
Soro	D1551	0	M
Soro	D1552	69	M
Soro	D1553	56	M
Soro	D1554	9	M
Soro	D1555	63	F
Soro	D1556	75	M
Soro	D1557	78	M
Soro	D1558	56	M
Soro	D1559	50	M
Soro	D1560	63	F
Soro	D1561	61	F
Soro	D1562	89	M
Soro	D1563	83	F
Soro	D1564	57	F
Soro	D1565	46	F
Soro	D1566	46	F
Soro	D1567	93	F
Soro	D1568	9	F
Soro	D1569	0	F
Soro	D1570	0	M
Soro	D1571	89	F
Soro	D1572	89	M
Soro	D1573	23	F
Soro	D1574	75	F
Soro	D1575	10	M
Soro	D1576	22	F
Soro	D1577	0	F
Soro	D1578	58	F
Soro	D1579	50	M
Soro	D1580	7	M
Soro	D1581	48	M
Soro	D1582	0	M
Soro	D1583	72	M
Soro	D1584	53	F
Soro	D1585	53	M
Soro	D1586	53	F
Soro	D1587	46	M
Liquor/LCR	D1588	55	M
Soro	D1589	60	F
Soro	D1590	38	F
Soro	D1591	55	F
Soro	D1592	44	M
Soro	D1593	21	F
Soro	D1594	55	M
Soro	D1595	45	F
Soro	D1596	56	M
Soro	D1597	31	M
Soro	D1598	42	M

Soro	D1599	70	F
Soro	D1600	39	F
Soro	D1601	42	F
Soro	D1602	0	I
Soro	D1603	0	I
Soro	D1604	0	I
Soro	D1605	0	M
Soro	D1606	0	F
Soro	D1607	56	F
Soro	D1608	70	M
Soro	D1609	73	F
Soro	D1610	56	M
Soro	D1611	44	M
Soro	D1612	49	M
Soro	D1613	0	M
Soro	D1614	0	M
Soro	D1615	19	F
Soro	D1616	6	M
Liquor/LCR	D1617	77	M
Soro	D1618	54	M
Liquor/LCR	D1619	0	F
Soro	D1620	0	M
Soro	D1621	0	M
Soro	D1622	49	F
Soro	D1623	40	M
Soro	D1624	46	F
Soro	D1625	61	F
Soro	D1626	45	M
Soro	D1627	40	F
Soro	D1628	95	F
Soro	D1629	67	M
Soro	D1630	58	M
Soro	D1631	41	M
Soro	D1632	14	F
Soro	D1633	88	M
Soro	D1634	0	M
Soro	D1635	40	I
Soro	D1636	73	M
Soro	D1637	0	F
Soro	D1638	56	F
Soro	D1639	54	F
Soro	D1640	75	M
Soro	D1641	53	M
Soro	D1642	82	M
Soro	D1643	71	M
Soro	D1644	39	M
Soro	D1645	41	M
Soro	D1646	28	M
Soro	D1647	54	F
Soro	D1648	63	F

Soro	D1649	53	F
Soro	D1650	78	M
Soro	D1651	40	M
Soro	D1652	62	F
Soro	D1653	57	M
Soro	D1654	59	F
Liquor/LCR	D1655	85	F
Soro	D1656	70	M
Soro	D1657	47	M
Soro	D1658	11	M
Soro	D1659	0	M
Soro	D1660	0	F
Soro	D1661	44	F
Soro	D1662	76	F
Soro	D1663	65	F
Soro	D1664	44	M
Soro	D1665	39	F
Soro	D1666	30	F
Soro	D1667	53	F
Soro	D1668	42	M
Soro	D1669	70	M
Soro	D1670	71	M
Soro	D1671	55	M
Soro	D1672	61	M
Soro	D1673	57	M
Soro	D1674	62	F
Soro	D1675	82	F
Soro	D1676	59	F
Soro	D1677	0	M
Soro	D1678	0	F
Soro	D1679	0	M
Soro	D1680	74	F
Soro	D1681	0	F
Soro	D1682	0	F
Soro	D1683	44	M
Soro	D1684	21	F
Soro	D1685	74	M
Soro	D1686	0	M
Soro	D1687	44	M
Soro	D1688	59	F
Soro	D1689	63	M
Soro	D1690	76	F
Soro	D1691	60	F
Soro	D1692	24	F
Soro	D1693	61	M
Soro	D1694	32	F
Soro	D1695	70	M
Soro	D1696	31	F
Soro	D1697	77	M
Soro	D1698	0	F

Soro	D1699	39	F
Soro	D1700	50	F
Soro	D1701	38	M
Soro	D1702	56	M
Soro	D1703	73	M
Liquor/LCR	D1704	44	M
Soro	D1705	37	M
Soro	D1706	54	M
Soro	D1707	59	M
Soro	D1708	90	F
Soro	D1709	47	F
Soro	D1710	49	M
Soro	D1711	73	M
Soro	D1712	34	F
Soro	D1713	7	M
Soro	D1714	51	M
Soro	D1715	44	M
Soro	D1716	25	F
Soro	D1717	44	M
Soro	D1718	71	F
Soro	D1719	69	F
Soro	D1720	33	F
Soro	D1721	30	F
Soro	D1722	36	F
Soro	D1723	45	F
Soro	D1724	26	M
Soro	D1725	77	F
Soro	D1726	0	M
Soro	D1727	47	M
Soro	D1728	45	M
Soro	D1729	78	F
Soro	D1730	61	M
Soro	D1731	36	F
Soro	D1732	52	F
Soro	D1733	45	M
Soro	D1734	35	F
Soro	D1735	79	F
Soro	D1736	77	M
Soro	D1737	60	F
Soro	D1738	30	F
Soro	D1739	0	F
Soro	D1740	0	F
Soro	D1741	0	M
Soro	D1742	55	F
Soro	D1743	15	F
Soro	D1744	0	F
Soro	D1745	41	F
Soro	D1746	68	F
Soro	D1747	61	M
Soro	D1748	59	F

Soro	D1749	0	F
Soro	D1750	41	M
Soro	D1751	29	M
Soro	D1752	48	F
Soro	D1753	51	F
Soro	D1754	65	M
Soro	D1755	29	M
Liquor/LCR	D1756	60	F
Soro	D1757	53	M
Soro	D1758	77	M
Soro	D1759	44	F
Soro	D1760	44	F
Soro	D1761	87	F
Soro	D1762	69	M
Soro	D1763	56	F
Soro	D1764	74	M
Soro	D1765	81	F
Soro	D1766	60	M
Soro	D1767	0	M
Soro	D1768	0	M
Soro	D1769	80	F
Soro	D1770	92	M
Soro	D1771	78	M
Soro	D1772	38	M
Soro	D1773	49	F
Soro	D1774	80	F
Soro	D1775	67	F
Soro	D1776	60	M
Soro	D1777	63	F
Soro	D1778	20	M
Soro	D1779	80	M
Soro	D1780	41	F
Soro	D1781	64	F
Soro	D1782	49	M
Soro	D1783	59	M
Soro	D1784	56	F
Soro	D1785	59	F
Soro	D1786	57	F
Soro	D1787	60	M
Soro	D1788	67	M
Soro	D1789	59	M
Soro	D1790	53	F
Soro	D1791	51	F
Soro	D1792	60	F
Soro	D1793	65	F
Soro	D1794	60	F
Soro	D1795	90	F
Soro	D1796	72	F
Soro	D1797	22	M
Soro	D1798	51	F

Soro	D1799	43	F
Soro	D1800	49	F
Soro	D1801	85	F
Soro	D1802	0	F
Soro	D1803	31	F
Soro	D1804	31	F
Soro	D1805	31	F
Soro	D1806	60	F
Soro	D1807	36	F
Soro	D1808	41	F
Soro	D1809	59	F
Soro	D1810	89	F
Liquor/LCR	D1811	0	M
Soro	D1812	89	M
Soro	D1813	36	M
Soro	D1814	49	M
Soro	D1815	46	F
Soro	D1816	61	F
Soro	D1817	73	F
Soro	D1818	63	F
Soro	D1819	80	M
Soro	D1820	33	M
Soro	D1821	0	M
Liquor/LCR	D1822	54	M
Soro	D1823	63	M
Soro	D1824	47	M
Liquor/LCR	D1825	50	M
Soro	D1826	82	M
Soro	D1827	39	M
Soro	D1828	41	M
Soro	D1829	72	M
Soro	D1830	56	M
Soro	D1831	72	M
Soro	D1832	76	F
Soro	D1833	71	M
Soro	D1834	40	M
Soro	D1835	87	F
Soro	D1836	68	M
Soro	D1837	64	M
Liquor/LCR	D1838	62	F
Soro	D1839	41	F
Soro	D1840	31	M
Soro	D1841	0	M
Soro	D1842	40	M
Soro	D1843	38	F
Soro	D1844	82	M
Soro	D1845	71	M
Soro	D1846	43	F
Liquor/LCR	D1847	84	M
Soro	D1848	26	F

Soro	D1849	9	M
Soro	D1850	73	F
Soro	D1851	83	F
Soro	D1852	59	F
Soro	D1853	26	M
Liquor/LCR	D1854	29	F
Soro	D1855	87	M
Soro	D1856	83	F
Liquor/LCR	D1857	27	F
Soro	D1858	19	M
Soro	D1859	42	F
Soro	D1860	21	F
Soro	D1861	46	F
Soro	D1862	87	M
Soro	D1863	81	M
Soro	D1864	57	M
Soro	D1865	48	M
Soro	D1866	91	M
Soro	D1867	34	M
Soro	D1868	0	F
Soro	D1869	0	F
Soro	D1870	0	M
Liquor/LCR	D1871	43	F
Soro	D1872	50	M
Soro	D1873	78	F
Soro	D1874	40	F
Soro	D1875	77	M
Soro	D1876	52	F
Soro	D1877	11	M
Soro	D1878	0	F
Soro	D1879	13	M
Soro	D1880	0	F
Soro	D1881	32	M
Soro	D1882	52	M
Liquor/LCR	D1883	66	M
Soro	D1884	66	F
Soro	D1885	45	M
Soro	D1886	86	M
Soro	D1887	75	M
Soro	D1888	23	F
Soro	D1889	34	M
Soro	D1890	21	M
Soro	D1891	34	M
Soro	D1892	35	M
Soro	D1893	34	M
Soro	D1894	84	M
Soro	D1895	70	M
Soro	D1896	92	M
Soro	D1897	62	F
Soro	D1898	94	F

Soro	D1899	33	F
Soro	D1900	33	F
Soro	D1901	82	M
Soro	D1902	77	F
Soro	D1903	0	F
Soro	D1904	9	M
Soro	D1905	56	M
Soro	D1906	59	M
Soro	D1907	49	M
Soro	D1908	69	M
Soro	D1909	69	M
Soro	D1910	24	M
Soro	D1911	36	M
Soro	D1912	0	F
Soro	D1913	0	F
Soro	D1914	0	F
Soro	D1915	71	M
Soro	D1916	64	F
Soro	D1917	63	M
Soro	D1918	36	F
Soro	D1919	63	F
Soro	D1920	68	M
Soro	D1921	38	M
Soro	D1922	58	F
Soro	D1923	0	M
Soro	D1924	68	F
Soro	D1925	87	M
Soro	D1926	64	F
Soro	D1927	68	F
Soro	D1928	67	I
Soro	D1929	62	M
Soro	D1930	85	M
Soro	D1931	71	M
Soro	D1932	38	M
Soro	D1933	43	M
Soro	D1934	59	F
Soro	D1935	79	M
Soro	D1936	49	F
Soro	D1937	68	M
Soro	D1938	32	M
Soro	D1939	53	F
Soro	D1940	11	M
Soro	D1941	77	F
Soro	D1942	69	F
Soro	D1943	72	F
Liquor/LCR	D1944	77	M
Soro	D1945	44	F
Soro	D1946	0	M
Soro	D1947	49	F
Soro	D1948	73	M

Soro	D1949	82	F
Soro	D1950	39	M
Soro	D1951	96	M
Soro	D1952	53	F
Soro	D1953	0	F
Soro	D1954	84	M
Soro	D1955	92	M
Soro	D1956	11	M
Soro	D1957	56	F
Soro	D1958	92	M
Soro	D1959	73	F
Soro	D1960	52	M
Soro	D1961	61	F
Soro	D1962	59	F
Soro	D1963	82	M
Soro	D1964	42	M
Soro	D1965	64	M
Soro	D1966	51	M
Soro	D1967	64	M
Soro	D1968	0	F
Soro	D1969	59	F
Soro	D1970	11	M
Liquor/LCR	D1971	76	F
Soro	D1972	56	M
Soro	D1973	41	F
Soro	D1974	63	M
Soro	D1975	78	M
Soro	D1976	72	F
Soro	D1977	88	M
Soro	D1978	64	F
Soro	D1979	19	M
Soro	D1980	49	F
Soro	D1981	31	F
Soro	D1982	70	F
Soro	D1983	47	M
Soro	D1984	31	F
Soro	D1985	55	M
Soro	D1986	43	F
Soro	D1987	44	F
Soro	D1988	51	F
Soro	D1989	69	M
Soro	D1990	57	F
Soro	D1991	39	F
Soro	D1992	20	F
Soro	D1993	49	M
Soro	D1994	35	F
Soro	D1995	6	M
Soro	D1996	63	M
Soro	D1997	63	M
Soro	D1998	42	F

Soro	D1999	54	F
Soro	D2000	79	F
Soro	D2001	27	M
Soro	D2002	23	M
Soro	D2003	14	M
Soro	D2004	0	M
Soro	D2005	64	M
Soro	D2006	89	M
Soro	D2007	64	M
Soro	D2008	59	F
Soro	D2009	0	F
Soro	D2010	29	M
Soro	D2011	70	F
Soro	D2012	48	M
Soro	D2013	53	M
Liquor/LCR	D2014	85	M
Soro	D2015	40	M
Soro	D2016	37	F
Soro	D2017	84	F
Soro	D2018	42	F
Soro	D2019	56	M
Soro	D2020	40	F
Soro	D2021	0	M
Soro	D2022	51	F
Soro	D2023	99	F
Soro	D2024	65	F
Soro	D2025	57	F
Soro	D2026	87	M
Soro	D2027	52	F
Soro	D2028	62	M
Soro	D2029	62	F
Soro	D2030	41	F
Soro	D2031	82	F
Soro	D2032	43	F
Soro	D2033	49	F
Soro	D2034	67	M
Soro	D2035	69	F
Soro	D2036	71	F
Soro	D2037	43	F
Soro	D2038	35	M
Soro	D2039	18	F
Soro	D2040	95	M
Soro	D2041	27	F
Soro	D2042	0	M
Liquor/LCR	D2043	40	M
Liquor/LCR	D2044	77	M
Soro	D2045	45	F
Soro	D2046	58	M
Soro	D2047	33	M
Soro	D2048	21	F

Soro	D2049	61	M
Soro	D2050	80	M
Soro	D2051	79	F
Soro	D2052	77	M
Soro	D2053	43	F
Soro	D2054	54	M
Liquor/LCR	D2055	46	M
Soro	D2056	65	M
Liquor/LCR	D2057	79	F
Soro	D2058	81	M
Soro	D2059	71	F
Soro	D2060	0	M
Soro	D2061	68	M
Soro	D2062	52	M
Soro	D2063	68	M
Soro	D2064	51	F
Soro	D2065	51	M
Soro	D2066	69	M
Soro	D2067	19	M
Soro	D2068	51	M
Soro	D2069	93	M
Soro	D2070	40	M
Soro	D2071	55	F
Soro	D2072	66	F
Soro	D2073	28	M
Soro	D2074	93	F
Soro	D2075	49	M
Soro	D2076	76	M
Soro	D2077	0	M
Soro	D2078	43	F
Soro	D2079	8	M
Soro	D2080	85	F
Soro	D2081	74	M
Soro	D2082	66	M
Soro	D2083	66	M
Liquor/LCR	D2084	44	F
Soro	D2085	37	F
Soro	D2086	80	F
Soro	D2087	62	M
Soro	D2088	61	F
Soro	D2089	61	M
Soro	D2090	33	M
Liquor/LCR	D2091	36	F
Soro	D2092	60	F
Soro	D2093	0	M
Soro	D2094	37	M
Soro	D2095	60	F
Soro	D2096	36	F
Soro	D2097	47	F
Soro	D2098	51	F

Soro	D2099	60	M
Soro	D2100	19	M
Soro	D2101	66	F
Soro	D2102	0	F
Liquor/LCR	D2103	82	F
Soro	D2104	71	M
Soro	D2105	68	M
Soro	D2106	85	M
Soro	D2107	45	F
Soro	D2108	47	F
Soro	D2109	47	F
Soro	D2110	56	M
Soro	D2111	58	F
Soro	D2112	61	M
Soro	D2113	50	F
Soro	D2114	75	M
Soro	D2115	41	M
Soro	D2116	76	F
Soro	D2117	59	F
Soro	D2118	50	M
Soro	D2119	60	F
Soro	D2120	74	M
Liquor/LCR	D2121	11	M
Soro	D2122	54	F
Soro	D2123	39	F
Liquor/LCR	D2124	50	M
Liquor/LCR	D2125	50	M
Soro	D2126	51	M
Soro	D2127	71	F
Soro	D2128	79	M
Soro	D2129	0	M
Soro	D2130	39	M
Soro	D2131	79	M
Soro	D2132	0	M
Soro	D2133	32	M
Soro	D2134	76	F
Soro	D2135	44	F
Soro	D2136	81	M
Soro	D2137	41	M
Soro	D2138	41	F
Soro	D2139	86	M
Soro	D2140	39	M
Soro	D2141	53	M
Soro	D2142	40	F
Soro	D2143	29	M
Soro	D2144	49	F
Soro	D2145	60	M
Soro	D2146	49	F
Soro	D2147	44	M
Soro	D2148	71	M

Soro	D2149	81	F
Soro	D2150	95	F
Soro	D2151	68	M
Soro	D2152	94	M
Soro	D2153	33	F
Soro	D2154	81	F
Soro	D2155	83	F
Soro	D2156	7	F
Soro	D2157	82	F
Soro	D2158	22	M
Soro	D2159	50	M
Soro	D2160	85	M
Soro	D2161	38	M
Soro	D2162	71	M
Soro	D2163	54	M
Soro	D2164	0	F
Soro	D2165	0	F
Soro	D2166	47	M
Soro	D2167	58	F
Soro	D2168	39	M
Soro	D2169	83	M
Soro	D2170	69	M
Soro	D2171	47	M
Soro	D2172	36	F
Soro	D2173	87	F
Soro	D2174	85	F
Soro	D2175	56	M
Soro	D2176	3	M
Soro	D2177	42	F
Soro	D2178	53	F
Soro	D2179	80	M
Soro	D2180	52	F
Soro	D2181	0	M
Soro	D2182	69	M
Soro	D2183	60	M
Liquor/LCR	D2184	80	F
Soro	D2185	48	F
Soro	D2186	59	M
Soro	D2187	61	M
Soro	D2188	77	M
Soro	D2189	34	F
Soro	D2190	31	M
Soro	D2191	62	F
Soro	D2192	66	M
Soro	D2193	69	F
Soro	D2194	70	M
Soro	D2195	66	F
Soro	D2196	20	M
Soro	D2197	90	M
Soro	D2198	31	M

Soro	D2199	48	M
Soro	D2200	82	F
Soro	D2201	80	M
Soro	D2202	80	M
Soro	D2203	68	F
Soro	D2204	67	F
Soro	D2205	93	F
Soro	D2206	0	M
Soro	D2207	0	M
Soro	D2208	71	F
Soro	D2209	34	M
Soro	D2210	45	F
Soro	D2211	48	M
Soro	D2212	64	M
Soro	D2213	56	F
Soro	D2214	39	F
Liquor/LCR	D2215	40	F
Soro	D2216	44	F
Soro	D2217	70	M
Liquor/LCR	D2218	44	F
Soro	D2219		M
Liquor/LCR	D2220		M
Soro	D2221	77	M
Soro	D2222	61	M
Soro	D2223	85	F
Liquor/LCR	D2224	42	F
Soro	D2225	39	F
Soro	D2226	44	M
Soro	D2227	39	F
Liquor/LCR	D2228	77	M
Soro	D2229		F
Soro	D2230	73	M
Soro	D2231	76	M
Soro	D2232	69	M
Soro	D2233	67	M
Soro	D2234	37	F
Soro	D2235	70	F
Soro	D2236	45	F
Soro	D2237	47	F
Soro	D2238	75	M
Soro	D2239	51	F
Soro	D2240	41	M
Soro	D2241	44	F
Soro	D2242		M
Soro	D2243	83	F
Liquor/LCR	D2244	30	M
Soro	D2245	49	F
Soro	D2246	62	F
Soro	D2247	85	M
Liquor/LCR	D2248	74	F

Soro	D2249	53	I
Soro	D2250		F
Soro	D2251	33	F
Soro	D2252	70	M
Soro	D2253	35	F
Soro	D2254	60	F
Soro	D2255	82	F
Liquor/LCR	D2256	62	M
Soro	D2257		M
Soro	D2258	50	F
Soro	D2259	74	M
Soro	D2260	32	F
Soro	D2261	22	M
Soro	D2262	36	M
Soro	D2263	39	M
Soro	D2264	40	M
Soro	D2265	48	M
Soro	D2266	64	M
Soro	D2267	81	F
Soro	D2268	57	F
Soro	D2269	32	M
Soro	D2270	49	F
Soro	D2271	33	M
Soro	D2272	59	F
Soro	D2273		M
Soro	D2274	43	F
Soro	D2275	57	M
Soro	D2276	57	M
Soro	D2277	66	M
Soro	D2278	57	M
Soro	D2279	68	M
Soro	D2280	48	F
Soro	D2281	85	F
Liquor/LCR	D2282	83	M
Soro	D2283	56	F
Soro	D2284	55	M
Soro	D2285	59	M
Soro	D2286	50	F
Soro	D2287	85	M
Soro	D2288	39	F
Soro	D2289	42	M
Soro	D2290	63	M
Soro	D2291	63	F
Soro	D2292	25	F
Soro	D2293	80	M
Soro	D2294		M
Soro	D2295		M
Soro	D2296	92	F
Soro	D2297	82	F
Soro	D2298	80	F

Soro	D2299	65	M
Soro	D2300	29	M
Soro	D2301	45	F
Liquor/LCR	D2302	48	M
Liquor/LCR	D2303	48	M
Soro	D2304	65	F
Soro	D2305	51	M
Soro	D2306		F
Soro	D2307	80	M
Liquor/LCR	D2308	36	F
Soro	D2309	81	M
Soro	D2310	67	F
Soro	D2311	46	M
Soro	D2312		M
Soro	D2313	62	M
Soro	D2314	63	M
Soro	D2315	47	F
Soro	D2316	46	M
Soro	D2317	27	M
Soro	D2318		F
Soro	D2319		M
Soro	D2320	43	F
Soro	D2321	87	M

Result
Negativo
Negativo
Negativo
Negativo
Negativo
Negativo
Negativo
Negativo
Positivo
Negativo
Negativo
Negativo
Negativo
Negativo
Negativo
Negativo
Negativo
Negativo
Negativo
Negativo
Negativo
Positivo
Negativo
Negativo
Negativo
Negativo
Negativo
Negativo
Negativo
Positivo
Negativo
Negativo
Negativo
Negativo
Negativo
Negativo
Negativo
Negativo
Negativo
Negativo
Negativo
Negativo
Negativo
Negativo
Negativo
Negativo
Negativo
Negativo

Negativo
Negativo
Negativo
Positivo
Negativo
Positivo
Negativo
Negativo
Negativo
Negativo
Negativo
Negativo
Negativo
Negativo
Negativo
Negativo
Negativo
Negativo
Negativo
Negativo
Negativo
Negativo
Negativo
Negativo
Negativo
Positivo
Negativo
Negativo
Negativo
Negativo
Negativo
Negativo
Negativo
Negativo
Negativo
Negativo
Negativo
Negativo
Negativo
Negativo
Positivo
Negativo
Negativo
Negativo
Negativo
Negativo
Negativo
Negativo
Negativo
Negativo

Negativo
Negativo
Negativo
Negativo
Negativo
Negativo
Negativo
Negativo
Negativo
Negativo
Negativo
Positivo
Negativo
Negativo
Negativo
Negativo
Positivo
Negativo
Negativo
Negativo
Negativo
Negativo
Negativo
Negativo
Negativo
Negativo
Positivo
Negativo
Negativo
Negativo
Negativo
Negativo
Negativo
Negativo
Negativo
Negativo
Negativo
Negativo
Negativo
Negativo
Negativo
Negativo
Negativo
Negativo
Negativo
Negativo
Negativo
Negativo
Negativo
Negativo

Negativo
Negativo
Negativo
Negativo
Negativo
Negativo
Negativo
Negativo
Negativo
Negativo
Negativo
Negativo
Negativo
Negativo
Negativo
Negativo
Negativo
Negativo
Negativo
Negativo
Negativo
Negativo
Positivo
Negativo
Negativo
Negativo
Negativo
Negativo
Positivo
Negativo
Negativo
Negativo
Negativo
Positivo
Negativo
Negativo
Negativo
Negativo
Negativo
Negativo
Negativo
Negativo
Negativo
Negativo
Negativo
Negativo
Negativo
Negativo
Negativo
Positivo

Negativo
Negativo
Negativo
Negativo
Negativo
Negativo
Negativo
Positivo
Positivo
Negativo
Negativo
Negativo
Negativo
Negativo
Negativo
Negativo
Negativo
Negativo
Negativo
Negativo
Negativo
Negativo
Negativo
Negativo
Negativo
Negativo
Negativo
Negativo
Positivo
Negativo
Negativo
Positivo
Negativo
Negativo
Negativo
Negativo
Negativo
Negativo
Negativo
Negativo
Negativo
Negativo
Negativo
Negativo
Positivo
Negativo
Negativo
Positivo
Negativo
Negativo

Positivo
Negativo
Negativo
Negativo
Negativo
Negativo
Negativo
Negativo
Negativo
Negativo
Negativo
Positivo
Positivo
Negativo
Negativo
Negativo
Negativo
Negativo
Negativo
Negativo
Negativo
Negativo
Negativo
Positivo
Negativo
Negativo
Positivo
Negativo
Negativo
Negativo
Negativo
Negativo
Negativo
Negativo
Positivo
Positivo
Negativo
Negativo
Negativo
Negativo
Positivo
Negativo
Negativo
Negativo
Negativo
Positivo
Negativo
Negativo
Negativo
Negativo

Negativo
Positivo
Negativo
Negativo
Negativo
Negativo
Negativo
Negativo
Negativo
Negativo
Negativo
Negativo
Negativo
Negativo
Negativo
Negativo
Negativo
Negativo
Negativo
Negativo
Negativo
Negativo
Negativo
Negativo
Negativo
Negativo
Negativo
Negativo
Negativo
Negativo
Negativo
Negativo
Negativo
Negativo
Negativo
Negativo
Negativo
Negativo
Negativo
Positivo
Negativo
Negativo
Negativo
Negativo
Negativo
Negativo
Negativo
Negativo
Negativo
Negativo

Negativo
Negativo
Negativo
Negativo
Negativo
Negativo
Negativo
Positivo
Negativo
Negativo
Negativo
Negativo
Negativo
Negativo
Negativo
Negativo
Negativo
Negativo
Negativo
Negativo
Negativo
Negativo
Negativo
Negativo
Negativo
Positivo
Negativo
Negativo
Negativo
Negativo
Negativo
Negativo
Negativo
Negativo
Negativo
Negativo
Negativo
Negativo
Negativo
Negativo
Negativo
Negativo
Positivo
Negativo
Negativo
Negativo
Negativo
Negativo
Negativo
Negativo

Negativo
Negativo
Negativo
Negativo
Positivo
Negativo
Negativo
Negativo
Negativo
Negativo
Positivo
Negativo
Negativo
Negativo
Negativo
Negativo
Negativo
Negativo
Negativo
Negativo
Negativo
Negativo
Negativo
Negativo
Negativo
Negativo
Negativo
Negativo
Negativo
Negativo
Negativo
Negativo
Negativo
Negativo
Negativo
Negativo
Positivo
Negativo
Negativo
Negativo
Negativo
Negativo
Negativo
Negativo
Negativo
Negativo
Negativo
Negativo
Negativo
Negativo

Negativo
Negativo
Negativo
Negativo
Negativo
Negativo
Negativo
Negativo
Negativo
Negativo
Negativo
Negativo
Negativo
Negativo
Negativo
Negativo
Negativo
Negativo
Negativo
Negativo
Negativo
Negativo
Negativo
Negativo
Negativo
Negativo
Negativo
Negativo
Negativo
Positivo
Negativo
Negativo
Negativo
Negativo
Negativo
Negativo
Negativo
Negativo
Positivo
Negativo
Negativo
Negativo
Negativo
Negativo
Negativo
Positivo
Negativo
Negativo
Negativo
Negativo

Negativo
Negativo
Positivo
Negativo
Negativo
Negativo
Negativo
Negativo
Negativo
Negativo
Negativo
Negativo
Negativo
Negativo
Negativo
Negativo
Negativo
Negativo
Negativo
Negativo
Negativo
Negativo
Negativo
Positivo
Negativo
Negativo
Negativo
Negativo
Positivo
Negativo
Negativo
Negativo
Negativo
Negativo
Negativo
Negativo
Negativo
Negativo
Negativo
Negativo
Negativo
Positivo
Positivo
Negativo
Positivo
Negativo
Negativo
Negativo
Negativo
Negativo

Negativo
Positivo
Negativo
Negativo
Negativo
Negativo
Negativo
Negativo
Positivo
Negativo
Negativo
Negativo
Negativo
Negativo
Negativo
Negativo
Negativo
Negativo
Negativo
Negativo
Negativo
Negativo
Negativo
Negativo
Negativo
Negativo
Negativo
Negativo
Negativo
Negativo
Positivo
Negativo
Negativo
Negativo
Negativo
Negativo
Negativo
Negativo
Negativo
Negativo
Negativo
Negativo
Positivo
Negativo
Negativo
Negativo
Negativo
Negativo
Negativo
Negativo

Positivo
Negativo
Negativo
Negativo
Negativo
Negativo
Negativo
Negativo
Negativo
Negativo
Negativo
Negativo
Negativo
Negativo
Positivo
Negativo
Negativo
Negativo
Negativo
Negativo
Negativo
Negativo
Negativo
Negativo
Negativo
Negativo
Negativo
Negativo
Negativo
Negativo
Negativo
Negativo
Negativo
Negativo
Negativo
Negativo
Negativo
Negativo
Negativo
Negativo
Negativo
Negativo
Negativo
Negativo
Negativo
Negativo
Negativo
Negativo
Negativo
Negativo

Negativo
Negativo
Positivo
Negativo
Negativo
Negativo
Negativo
Negativo
Negativo
Negativo
Negativo
Negativo
Negativo
Negativo
Negativo
Negativo
Negativo
Negativo
Negativo
Negativo
Negativo
Negativo
Negativo
Negativo
Negativo
Negativo
Negativo
Negativo
Negativo
Negativo
Negativo
Negativo
Negativo
Negativo
Positivo
Negativo
Negativo
Negativo
Negativo
Negativo
Negativo
Negativo
Negativo
Negativo
Negativo
Negativo
Negativo
Negativo
Negativo
Negativo

Negativo
Negativo
Negativo
Negativo
Negativo
Negativo
Positivo
Negativo
Negativo
Negativo
Negativo
Negativo
Negativo
Negativo
Negativo
Negativo
Positivo
Negativo
Negativo
Negativo
Negativo
Negativo
Negativo
Negativo
Negativo
Negativo
Negativo
Negativo
Negativo
Negativo
Negativo
Negativo
Negativo
Negativo
Negativo
Negativo
Negativo
Negativo
Negativo
Negativo
Negativo
Negativo
Negativo
Negativo
Negativo
Negativo
Negativo
Negativo
Negativo
Negativo

Negativo
Negativo
Positivo
Negativo
Negativo
Negativo
Negativo
Negativo
Negativo
Negativo
Negativo
Positivo
Negativo
Negativo
Negativo
Negativo
Negativo
Negativo
Negativo
Negativo
Negativo
Negativo
Negativo
Negativo
Negativo
Negativo
Negativo
Negativo
Negativo
Negativo
Negativo
Negativo
Negativo
Negativo
Negativo
Positivo
Negativo
Negativo
Negativo
Negativo
Negativo
Negativo
Negativo
Positivo
Negativo
Negativo
Positivo
Negativo
Negativo
Negativo

Negativo
Negativo
Negativo
Negativo
Negativo
Negativo
Negativo
Negativo
Negativo
Positivo
Negativo
Negativo
Positivo
Negativo
Negativo
Negativo
Negativo
Negativo
Negativo
Negativo
Negativo
Negativo
Negativo
Negativo
Negativo
Negativo
Negativo
Negativo
Negativo
Negativo
Negativo
Negativo
Negativo
Negativo
Negativo
Negativo
Negativo
Negativo
Negativo
Negativo
Negativo
Negativo
Negativo
Negativo
Positivo
Negativo
Negativo
Negativo
Negativo
Negativo

Negativo
Negativo
Negativo
Positivo
Negativo
Negativo
Negativo
Negativo
Negativo
Negativo
Negativo
Negativo
Negativo
Negativo
Negativo
Negativo
Negativo
Negativo
Negativo
Negativo
Negativo
Positivo
Negativo
Negativo
Negativo
Negativo
Negativo
Negativo
Positivo
Negativo
Negativo
Negativo
Negativo
Negativo
Negativo
Negativo
Negativo
Negativo
Negativo
Negativo
Negativo
Negativo
Negativo
Negativo
Negativo
Negativo
Negativo
Negativo
Negativo
Negativo

Negativo
Negativo
Negativo
Negativo
Negativo
Negativo
Negativo
Negativo
Negativo
Negativo
Negativo
Negativo
Negativo
Positivo
Negativo
Negativo
Negativo
Negativo
Negativo
Negativo
Negativo
Negativo
Negativo
Negativo
Negativo
Negativo
Negativo
Negativo
Negativo
Negativo
Negativo
Negativo
Negativo
Negativo
Negativo
Negativo
Negativo
Negativo
Negativo
Negativo
Negativo
Negativo
Negativo
Negativo
Negativo
Negativo
Negativo
Negativo
Negativo
Negativo

Negativo
Negativo
Negativo
Negativo
Negativo
Negativo
Negativo
Negativo
Negativo
Negativo
Negativo
Negativo
Negativo
Negativo
Negativo
Negativo
Negativo
Negativo
Negativo
Negativo
Negativo
Negativo
Negativo
Negativo
Negativo
Negativo
Negativo
Negativo
Negativo
Negativo
Negativo
Negativo
Negativo
Negativo
Negativo
Negativo
Negativo
Negativo
Negativo
Negativo
Negativo
Negativo
Negativo
Negativo
Negativo
Negativo
Negativo
Negativo
Negativo
Negativo

Negativo
Negativo
Negativo
Negativo
Negativo
Positivo
Negativo
Negativo
Negativo
Negativo
Negativo
Negativo
Negativo
Negativo
Negativo
Negativo
Negativo
Negativo
Negativo
Negativo
Negativo
Negativo
Negativo
Negativo
Negativo
Negativo
Negativo
Negativo
Negativo
Negativo
Negativo
Negativo
Negativo
Negativo
Negativo
Negativo
Negativo
Negativo
Negativo
Negativo
Negativo
Negativo
Negativo
Negativo
Negativo
Negativo
Negativo
Negativo
Negativo
Positivo

Negativo
Positivo
Negativo
Negativo
Positivo
Negativo
Negativo
Negativo
Negativo
Negativo
Negativo
Positivo
Negativo
Negativo
Negativo
Negativo
Negativo
Negativo
Negativo
Negativo
Negativo
Positivo
Positivo
Negativo
Negativo
Negativo
Negativo
Negativo
Negativo
Positivo
Positivo
Negativo
Negativo
Negativo
Negativo
Negativo
Negativo
Negativo
Negativo
Negativo
Negativo
Positivo
Negativo
Negativo
Negativo
Negativo
Negativo
Negativo
Negativo
Negativo

Negativo
Negativo
Negativo
Negativo
Negativo
Negativo
Positivo
Positivo
Positivo
Negativo
Negativo
Negativo
Negativo
Negativo
Negativo
Negativo
Negativo
Negativo
Negativo
Negativo
Positivo
Negativo
Negativo
Negativo
Negativo
Negativo
Negativo
Negativo
Negativo
Negativo
Negativo
Negativo
Negativo
Negativo
Negativo
Negativo
Negativo
Negativo
Negativo
Negativo
Negativo
Negativo
Negativo
Negativo
Negativo
Negativo
Negativo
Negativo
Negativo
Negativo

Negativo
Positivo
Negativo
Negativo
Negativo
Negativo
Positivo
Negativo
Negativo
Negativo
Negativo
Negativo
Negativo
Negativo
Negativo
Negativo (ver observações)
Negativo
Negativo
Negativo
Negativo
Negativo
Negativo
Negativo
Negativo
Negativo
Negativo
Negativo
Negativo
Negativo
Negativo
Negativo
Negativo
Negativo
Positivo
Positivo
Negativo
Negativo
Positivo
Negativo
Negativo
Negativo
Negativo
Negativo
Negativo
Negativo
Negativo
Negativo
Negativo
Negativo
Negativo

Negativo
Negativo
Negativo
Negativo
Negativo
Negativo
Negativo
Negativo
Negativo
Negativo
Negativo
Negativo
Positivo
Negativo
Negativo
Negativo
Negativo
Negativo
Negativo
Negativo
Negativo
Positivo
Positivo
Negativo
Negativo
Positivo
Negativo
Negativo
Negativo
Positivo
Positivo
Negativo
Positivo
Negativo
Negativo
Negativo
Positivo
Negativo
Negativo
Negativo
Negativo
Negativo
Negativo
Negativo
Negativo
Negativo
Negativo
Negativo
Negativo
Negativo

Negativo
Negativo
Positivo
Negativo
Negativo
Negativo
Negativo
Negativo
Negativo
Positivo
Negativo
Negativo
Negativo
Negativo
Negativo
Negativo
Negativo
Negativo
Negativo
Negativo
Negativo
Negativo
Negativo
Negativo
Negativo
Negativo
Negativo
Negativo
Positivo
Negativo
Negativo
Negativo
Negativo
Negativo
Negativo
Negativo
Negativo
Negativo
Negativo
Negativo
Negativo
Negativo
Negativo
Negativo
Negativo
Negativo
Positivo
Negativo
Negativo
Negativo

Negativo
Negativo
Negativo
Negativo
Negativo
Negativo
Negativo
Positivo
Negativo
Negativo
Negativo
Negativo
Negativo
Negativo
Negativo
Negativo
Negativo
Negativo
Negativo
Positivo
Negativo
Negativo
Positivo
Negativo
Negativo
Negativo
Negativo
Negativo
Negativo
Negativo
Negativo
Negativo
Negativo
Negativo
Negativo
Negativo
Negativo
Negativo
Negativo
Negativo
Positivo
Negativo
Negativo
Negativo
Positivo
Negativo
Negativo
Negativo
Negativo
Negativo

Negativo
Negativo
Positivo
Negativo
Negativo
Negativo
Negativo
Negativo
Negativo
Negativo
Negativo
Negativo
Negativo
Negativo
Negativo
Negativo
Negativo
Negativo
Negativo
Negativo
Negativo
Negativo
Negativo
Negativo
Negativo
Negativo
Negativo
Negativo
Negativo
Negativo
Negativo
Negativo
Negativo
Negativo
Negativo
Negativo
Negativo
Positivo
Negativo
Negativo
Negativo
Negativo
Negativo
Negativo
Negativo
Negativo
Negativo
Negativo
Negativo
Negativo

Negativo
Positivo
Negativo
Negativo
Negativo
Negativo
Negativo
Negativo
Negativo
Negativo
Negativo
Negativo
Negativo
Negativo
Negativo
Negativo
Negativo
Negativo
Negativo
Negativo
Negativo
Negativo
Negativo
Negativo
Negativo
Negativo
Negativo
Negativo
Negativo
Negativo
Negativo
Negativo
Negativo
Negativo
Negativo
Negativo
Negativo
Negativo
Negativo
Negativo
Negativo
Negativo
Negativo
Negativo
Negativo
Negativo
Negativo
Negativo
Negativo
Positivo

Negativo
Negativo
Negativo
Negativo
Negativo
Negativo
Negativo
Negativo
Negativo
Negativo
Negativo
Negativo
Negativo
Negativo
Negativo
Negativo
Negativo
Negativo
Negativo
Negativo
Negativo
Negativo
Negativo
Negativo
Negativo
Negativo
Negativo
Positivo
Negativo
Negativo
Negativo
Negativo
Negativo
Negativo
Negativo
Negativo
Negativo
Negativo
Negativo
Negativo
Negativo
Negativo
Negativo
Negativo
Positivo
Negativo
Negativo
Negativo
Negativo
Negativo

Negativo
Negativo
Negativo
Negativo
Negativo
Negativo
Negativo
Negativo
Negativo
Negativo
Negativo
Negativo
Negativo
Negativo
Negativo
Negativo
Negativo
Negativo
Positivo
Negativo
Negativo
Negativo
Negativo
Negativo
Positivo
Negativo
Negativo
Negativo
Negativo
Negativo
Negativo
Negativo
Negativo
Negativo
Negativo
Negativo
Negativo
Positivo
Negativo
Negativo
Negativo
Negativo
Negativo
Negativo
Negativo
Negativo
Negativo
Negativo
Negativo
Negativo

Negativo
Negativo
Negativo
Negativo
Negativo
Negativo
Negativo
Negativo
Negativo
Positivo
Negativo
Negativo
Negativo
Positivo
Positivo
Negativo
Negativo
Negativo
Negativo
Negativo
Negativo
Positivo
Negativo
Negativo
Negativo
Positivo
Negativo
Negativo
Negativo
Negativo
Positivo
Negativo
Negativo
Negativo
Negativo
Negativo
Negativo
Negativo
Negativo
Negativo
Negativo
Negativo
Negativo
Negativo
Negativo
Negativo
Negativo
Negativo
Negativo
Negativo

Negativo
Negativo
Negativo
Negativo
Negativo
Negativo
Negativo
Negativo
Negativo
Negativo
Negativo
Negativo
Negativo
Negativo
Positivo
Negativo
Negativo
Negativo
Negativo
Negativo
Negativo
Negativo
Negativo
Negativo
Negativo
Negativo
Negativo
Negativo
Negativo
Negativo
Negativo
Negativo
Negativo
Negativo
Negativo
Negativo
Negativo
Negativo
Negativo
Negativo
Negativo
Positivo
Negativo
Negativo
Negativo
Negativo
Negativo
Negativo
Negativo
Negativo

Negativo
Negativo
Negativo
Negativo
Negativo
Negativo
Negativo
Negativo
Negativo
Negativo
Negativo
Negativo
Positivo
Negativo
Negativo
Negativo
Negativo
Negativo
Negativo
Negativo
Negativo
Negativo
Negativo
Negativo
Negativo
Negativo
Negativo
Negativo
Negativo
Negativo
Negativo
Negativo
Negativo
Negativo
Negativo
Negativo
Negativo
Negativo
Negativo
Negativo
Positivo
Negativo
Negativo
Negativo
Positivo
Negativo
Negativo
Negativo
Negativo
Negativo

Negativo
Negativo
Negativo
Negativo
Negativo
Negativo
Negativo
Negativo
Negativo
Negativo
Negativo
Negativo
Negativo
Negativo
Negativo
Negativo
Negativo
Negativo
Negativo
Negativo
Negativo
Negativo
Negativo
Negativo
Negativo
Negativo
Negativo
Negativo
Negativo
Negativo
Negativo
Negativo
Negativo
Negativo
Negativo
Negativo
Negativo
Negativo
Negativo
Negativo
Negativo
Negativo
Negativo
Negativo
Negativo
Negativo
Negativo
Negativo
Negativo
Negativo

Negativo
Negativo
Positivo
Negativo
Negativo
Negativo
Positivo
Negativo
Negativo
Negativo
Negativo
Negativo
Negativo
Negativo
Negativo
Negativo
Negativo
Negativo
Negativo
Negativo
Negativo
Negativo
Negativo
Negativo
Negativo
Negativo
Negativo
Negativo
Negativo
Negativo
Negativo
Negativo
Negativo
Negativo
Negativo
Negativo
Negativo
Negativo
Negativo
Negativo
Negativo
Negativo
Negativo
Negativo
Negativo
Negativo
Negativo
Negativo
Negativo
Negativo

Negativo
Negativo
Negativo
Negativo
Negativo
Negativo
Negativo
Negativo
Negativo
Negativo
Negativo
Negativo
Negativo
Negativo
Negativo
Negativo
Negativo
Negativo
Positivo
Negativo
Negativo
Negativo
Negativo
Negativo
Negativo
Negativo
Negativo
Negativo
Negativo
Negativo
Negativo
Negativo
Negativo
Negativo
Negativo
Negativo
Negativo
Negativo
Negativo
Negativo
Negativo
Negativo
Negativo
Negativo
Negativo
Negativo
Negativo
Negativo
Negativo
Negativo

Negativo
Negativo
Negativo
Negativo
Negativo
Negativo
Negativo
Negativo
Negativo
Positivo
Negativo
Negativo
Negativo
Negativo
Negativo
Negativo
Negativo
Negativo
Negativo
Negativo
Negativo
Negativo
Negativo
Negativo
Negativo
Negativo
Negativo
Negativo
Positivo
Negativo
Negativo
Negativo
Positivo
Negativo
Negativo
Negativo
Negativo
Negativo
Negativo
Negativo
Negativo
Negativo
Negativo
Negativo
Negativo
Negativo
Negativo
Negativo
Negativo
Negativo

Negativo
Negativo
Negativo
Negativo
Negativo
Negativo
Negativo
Negativo
Negativo
Negativo
Negativo
Negativo
Negativo
Negativo
Negativo
Negativo
Negativo
Negativo
Negativo
Negativo
Negativo
Negativo
Positivo
Negativo
Negativo
Negativo
Negativo
Negativo
Negativo
Negativo
Negativo
Negativo
Negativo
Negativo
Negativo
Negativo
Negativo
Negativo
Negativo
Negativo
Negativo
Negativo
Negativo
Negativo
Negativo
Negativo
Negativo
Negativo
Negativo
Positivo

Negativo
Negativo
Negativo
Negativo
Negativo
Negativo
Negativo
Negativo
Negativo
Negativo
Positivo
Negativo
Negativo
Negativo
Negativo
Negativo
Negativo
Negativo
Negativo
Negativo
Negativo
Positivo
Negativo
Negativo
Negativo
Negativo
Negativo
Negativo
Negativo
Negativo
Positivo
Positivo
Negativo
Negativo
Negativo
Negativo
Negativo
Negativo
Negativo
Negativo
Negativo
Negativo
Negativo
Negativo
Negativo
Negativo
Negativo
Positivo
Negativo
Negativo

Negativo
Negativo
Negativo
Negativo
Negativo
Negativo
Negativo
Negativo
Negativo
Negativo
Negativo
Negativo
Negativo
Negativo
Negativo
Negativo
Positivo
Negativo
Negativo
Negativo
Negativo
Negativo
Negativo
Negativo
Negativo
Negativo
Negativo
Negativo
Negativo
Negativo
Negativo
Negativo
Negativo
Negativo
Positivo
Negativo
Negativo
Negativo
Negativo
Negativo
Negativo
Negativo
Negativo
Negativo
Negativo
Negativo
Negativo
Negativo
Negativo
Negativo

Negativo
Negativo
Negativo
Negativo
Negativo
Negativo
Negativo
Negativo
Negativo
Negativo
Negativo
Positivo
Negativo
Negativo
Negativo
Negativo
Negativo
Negativo
Positivo
Negativo
Negativo
Negativo
Negativo
Negativo
Negativo
Negativo
Negativo
Positivo
Negativo
Negativo
Negativo
Negativo
Positivo
Negativo
Negativo
Negativo
Negativo
Negativo
Negativo
Negativo
Negativo
Negativo
Negativo
Negativo
Negativo
Negativo
Negativo
Negativo
Negativo
Negativo

Positivo
Negativo
Negativo
Negativo
Negativo
Negativo
Negativo
Negativo
Negativo
Negativo
Negativo
Negativo
Negativo
Negativo
Negativo
Negativo
Negativo
Positivo
Positivo
Negativo
Negativo
Positivo
Negativo
Negativo
Positivo
Negativo
Negativo
Negativo
Negativo
Negativo
Negativo
Negativo
Negativo
Negativo
Negativo
Negativo
Negativo
Negativo
Negativo
Negativo
Negativo
Negativo
Positivo
Negativo
Positivo
Negativo
Negativo
Negativo
Negativo
Negativo

Negativo
Negativo
Negativo
Negativo
Negativo
Negativo
Negativo
Negativo
Negativo
Negativo
Positivo
Negativo
Negativo
Negativo
Negativo
Negativo
Negativo
Positivo
Negativo
Negativo
Negativo
Positivo
Positivo
Negativo
Negativo
Negativo
Negativo
Negativo
Negativo
Negativo
Positivo
Negativo
Negativo
Positivo
Negativo
Negativo
Negativo
Negativo
Negativo
Positivo
Negativo
Negativo
Positivo
Negativo
Negativo
Negativo
Negativo
Negativo
Negativo
Negativo

Negativo
Negativo
Negativo
Negativo
Negativo
Negativo
Negativo
Positivo
Positivo
Negativo
Negativo
Negativo
Negativo
Negativo
Negativo
Positivo
Negativo
Negativo
Negativo
Negativo
Positivo
Negativo
Negativo
Negativo
Negativo
Negativo
Negativo
Positivo
Negativo
Negativo
Negativo
Negativo
Negativo
Negativo
Negativo
Negativo
Negativo
Negativo
Positivo
Negativo
Negativo
Positivo
Negativo
Negativo
Negativo
Negativo
Negativo
Negativo
Negativo
Negativo

Negativo
Negativo
Negativo
Negativo
Negativo
Negativo
Negativo
Negativo
Negativo
Negativo
Negativo
Negativo
Negativo
Positivo
Negativo
Negativo
Negativo
Negativo
Negativo
Negativo
Negativo
Negativo
Negativo
Negativo
Negativo
Negativo
Negativo
Negativo
Negativo
Negativo
Negativo
Negativo
Negativo
Negativo
Negativo
Negativo
Negativo
Negativo
Negativo
Negativo
Negativo
Negativo
Negativo
Negativo
Negativo
Negativo
Negativo
Negativo
Negativo
Negativo

Negativo
Negativo
Negativo
Negativo
Negativo
Negativo
Negativo
Negativo
Negativo
Negativo
Negativo
Negativo
Negativo
Positivo
Negativo
Negativo
Negativo
Negativo
Negativo
Negativo
Negativo
Negativo
Negativo

Data of molecular biology

Type of sample	Patient	Age	Sex	Result
Hemocultura	B1	57	M	Negativo
Sangue	B2	54	M	Negativo
Liquor/LCR	B3	35	F	Negativo
Sangue	B4	35	F	Negativo
Sangue	B5	67	F	Negativo
Sangue	B6	74	M	Negativo
Hemocultura	B7	11	M	Brucella melitensis
Liquor/LCR	B8	42	F	Negativo
Estirpe	B9	72	M	Brucella melitensis
Liquor/LCR	B10	0	M	Negativo
Sangue	B11	82	F	Negativo
Sangue	B12	67	F	Negativo
Liquor/LCR	B13	67	F	Negativo
Sangue	B14	67	F	Negativo
Liquor/LCR	B15	46	M	Negativo
Sangue	B16	82	F	Negativo
Liquor/LCR	B17	76	M	Negativo
Liquor/LCR	B18	81	M	Negativo
Liquor/LCR	B19	62	M	Negativo
Liquor/LCR	B20	61	M	Negativo
Sangue	B21	61	M	Negativo
Sangue	B22	71	F	Negativo
Sangue	B23	71	F	Negativo
Biopsia	B24	34	F	Negativo
Sangue	B25	74	M	Negativo
Biopsia	B26	18	F	Negativo
Liquor/LCR	B27	18	F	Negativo
Sangue	B28	14	M	Negativo
Liquor/LCR	B29	41	M	Negativo
Sangue	B30	17	F	Negativo
Estirpe	B31	53	M	Brucella melitensis
Liquor/LCR	B32	64	M	Negativo
Liquor/LCR	B33	73	M	Negativo
Estirpe	B34	0	F	Brucella melitensis
Hemocultura	B35	46	M	Negativo
Sangue	B36	20	F	Negativo
Estirpe	B37	47	F	Brucella melitensis
Biopsia	B38	68	F	Negativo
Sangue	B39	68	F	Negativo
Sangue	B40	74	M	Negativo
Biopsia	B41	76	F	Negativo
Liquor/LCR	B42	65	M	Negativo
Hemocultura	B43	54	M	Brucella melitensis
Sangue	B44	48	F	Negativo
Sangue	B45	76	F	Negativo
Sangue	B46	72	M	Negativo
Hemocultura	B47	30	F	Negativo
Sangue	B48	63	M	Negativo

Sangue	B49	30	F	Negativo
Sangue	B50	49	F	Negativo
Sangue	B51	30	F	Negativo
Sangue	B52	80	M	Negativo
Sangue	B53	52	M	Negativo
Sangue	B54	41	M	Negativo
Sangue	B55	30	F	Negativo
Sangue	B56	65	F	Negativo
Liquor/LCR	B57	70	F	Negativo
Hemocultura	B58	84	F	Negativo
Liquor/LCR	B59	53	M	Negativo
Liquor/LCR	B60	56	M	Negativo
Sangue	B61	56	M	Negativo
Liquor/LCR	B62	58	M	Negativo
Estirpe	B63	87	M	Brucella melitensis
Liquor/LCR	B64	0	F	Negativo
Biopsia	B65	68	M	Brucella melitensis
Sangue	B66	68	M	Negativo
Sangue	B67	61	F	Negativo
Liquor/LCR	B68	88	F	Negativo
Liquor/LCR	B69	61	M	Negativo
Liquor/LCR	B70	82	F	Brucella melitensis
Sangue	B71	53	F	Negativo
Sangue	B72	76	F	Negativo
Sangue	B73	40	F	Negativo
Liquor/LCR	B74	83	M	Negativo
Sangue	B75	74	M	Negativo
Sangue	B76	41	M	Negativo
Sangue	B77	54	F	Negativo
Sangue	B78	52	M	Negativo
Sangue	B79	63	M	Brucella melitensis
Sangue	B80	40	M	Negativo
Liquor/LCR	B81	66	M	Negativo
Sangue	B82	73	M	Negativo
Liquor/LCR	B83	65	F	Negativo
Liquor/LCR	B84	56	M	Negativo
Estirpe	B85	61	M	Negativo
Liquor/LCR	B86	70	M	Negativo
Sangue	B87	36	M	Negativo
Liquor/LCR	B88	36	M	Negativo
Liquor/LCR	B89	31	F	Negativo
Biopsia	B90	54	M	Negativo
Sangue	B91	46	M	Negativo
Liquor/LCR	B92	49	F	Negativo
Estirpe	B93	50	F	Brucella melitensis
Sangue	B94	41	F	Negativo
Sangue	B95	8	F	Negativo
Sangue	B96	4	F	Negativo
Liquor/LCR	B97	81	M	Negativo
Biopsia	B98	64	M	Negativo

Sangue	B99	18	M	Negativo
Biopsia	B100	59	I	Negativo
Biopsia	B101	59	I	Negativo
Sangue	B102	5	F	Negativo
Sangue	B103	5	F	Negativo
Liquor/LCR	B104	50	M	Negativo
Biopsia	B105	19	M	Negativo
Sangue	B106	19	F	Negativo
Liquor/LCR	B107	45	M	Negativo
Liquor/LCR	B108	51	F	Negativo
Sangue	B109	38	F	Negativo
Liquor/LCR	B110	77	M	Negativo
Liquor/LCR	B111	52	M	Negativo
Liquor/LCR	B112	89	F	Negativo
Liquor/LCR	B113	67	M	Negativo
Sangue	B114	31	M	Negativo
Liquor/LCR	B115	37	F	Negativo
Liquor/LCR	B116	55	M	Negativo
Liquor/LCR	B117	48	F	Negativo
Exsudado	B118	0	M	Brucella melitensis
Sangue	B119	0	M	Brucella melitensis
Exsudado	B120	0	M	Brucella melitensis
Sangue	B121	0	M	Brucella melitensis
Exsudado	B122	0	M	Brucella melitensis
Sangue	B123	0	M	Brucella melitensis
Sangue	B124	0	M	Brucella melitensis
Exsudado	B125	0	M	Brucella melitensis
Exsudado	B126	0	M	Brucella melitensis
Sangue	B127	0	M	Brucella melitensis
Sangue	B128	0	M	Brucella melitensis
Exsudado	B129	0	M	Brucella melitensis
Sangue	B130	0	M	Brucella melitensis
Exsudado	B131	0	M	Brucella melitensis
Sangue	B132	0	M	Brucella melitensis
Exsudado	B133	0	M	Brucella melitensis
Sangue	B134	17	F	Negativo
Liquor/LCR	B135	79	F	Negativo
Liquor/LCR	B136	52	M	Negativo
Liquor/LCR	B137	33	M	Negativo
Liquor/LCR	B138	29	M	Negativo
Liquor/LCR	B139	55	F	Negativo
Liquor/LCR	B140	82	F	Negativo
Sangue	B141	19	M	Negativo
Liquor/LCR	B142	33	F	Negativo
Liquor/LCR	B143	83	M	Negativo
Liquor/LCR	B144	40	M	Negativo
Liquor/LCR	B145	55	F	Negativo
Liquor/LCR	B146	46	M	Negativo
Sangue	B147	43	F	Negativo
Liquor/LCR	B148	43	F	Negativo

Liquor/LCR	B149	75	M	Negativo
Liquor/LCR	B150	71	F	Negativo
Liquor/LCR	B151	78	F	Negativo
Liquor/LCR	B152	32	M	Negativo
Liquor/LCR	B153	85	F	Negativo
Liquor/LCR	B154	61	F	Negativo
Liquor/LCR	B155	71	F	Negativo
Liquor/LCR	B156	27	F	Negativo
Liquor/LCR	B157	68	M	Negativo
Liquor/LCR	B158	32	M	Negativo
Liquor/LCR	B159	62	M	Negativo
Sangue	B160	74	M	Negativo
Liquor/LCR	B161	48	M	Negativo
Liquor/LCR	B162	66	M	Negativo
Liquor/LCR	B163	91	M	Negativo
Liquor/LCR	B164	25	M	Negativo
Sangue	B165	25	M	Negativo
Liquor/LCR	B166	66	M	Negativo
Liquor/LCR	B167	80	M	Negativo
Liquor/LCR	B168	35	F	Negativo
Liquor/LCR	B169	71	F	negativo
Liquor/LCR	B170	75	M	Negativo
Liquor/LCR	B171	77	M	Negativo
Liquor/LCR	B172	81	M	Negativo
Liquor/LCR	B173	64	F	Negativo
Liquor/LCR	B174	68	M	Negativo
Biopsia	B175	60	M	Negativo
Liquor/LCR	B176	39	M	Negativo
Liquor/LCR	B177	75	M	Negativo
Liquor/LCR	B178	30	M	Negativo
Sangue	B179	30	M	Negativo
Liquor/LCR	B180	51	F	Negativo
Liquor/LCR	B181	51	F	Negativo
Liquor/LCR	B182	69	M	Negativo
Liquor/LCR	B183	81	F	Negativo
Liquor/LCR	B184	45	M	Negativo
Liquor/LCR	B185	62	F	Negativo
Sangue	B186	16	F	Negativo
Liquor/LCR	B187	81	M	Negativo
Liquor/LCR	B188	66	M	Negativo
Liquor/LCR	B189	26	M	Negativo
Liquor/LCR	B190	60	M	Negativo
Liquor/LCR	B191	50	F	Negativo
Liquor/LCR	B192	61	M	Negativo
Liquor/LCR	B193	67	F	Negativo
Liquor/LCR	B194	75	F	Negativo
Liquor/LCR	B195	88	M	Negativo
Liquor/LCR	B196	82	F	Negativo
Sangue	B197	66	M	Negativo
Liquor/LCR	B198	66	M	Negativo

Liquor/LCR	B199	37	M	Negativo
Biopsia	B200	51	M	Brucella melitensis
Liquor/LCR	B201	54	F	Negativo
Estirpe	B202	57	F	Negativo
Liquor/LCR	B203	33	F	Negativo
Liquor/LCR	B204	86	M	Negativo
Liquor/LCR	B205	84	F	Negativo
Liquor/LCR	B206	80	M	Negativo
Liquor/LCR	B207	45	M	Negativo
Liquor/LCR	B208	79	M	Negativo
Liquor/LCR	B209	73	F	Negativo
Sangue	B210	50	I	Negativo
Sangue	B211	82	I	Negativo
Biopsia	B212	83	I	Brucella melitensis
Sangue	B213	77	I	Negativo
Estirpe	B214	67	I	Brucella melitensis
Estirpe	B215	9	I	Brucella melitensis
Estirpe	B216	41	I	brucella melitensis
Estirpe	B217	43	I	brucella melitensis
Estirpe	B218	64	I	Brucella melitensis
Estirpe	B219	55	I	Brucella melitensis
Estirpe	B220	52	M	Brucella melitensis
Liquor/LCR	B221	75	M	Negativo
Estirpe	B222	72	F	Brucella melitensis
Estirpe	B223	14	M	Brucella melitensis
Liquor/LCR	B224	55	M	Negativo
Estirpe	B225	46	F	Brucella melitensis
Sangue	B226	15	M	Negativo
Sangue	B227	23	F	Negativo
Liquor/LCR	B228	23	F	Negativo
Liquor/LCR	B229	69	M	Negativo
Liquido sinovial	B230	46	M	Negativo
Sangue	B231	17	M	Negativo
Liquor/LCR	B232	17	M	Negativo
Liquor/LCR	B233	61	M	Negativo
Liquor/LCR	B234	86	M	Negativo
Liquor/LCR	B235	56	M	Negativo
Sangue	B236	90	F	Negativo
Biopsia	B237	59	F	Negativo
Sangue	B238	20	M	Negativo
Sangue	B239	59	F	Negativo
Sangue	B240	49	M	Negativo
Liquor/LCR	B241	27	F	Negativo
Sangue	B242	21	F	Negativo
Sangue	B243	78	F	Negativo
Liquor/LCR	B244	11	M	Negativo
Estirpe	B245	64	M	Brucella mellitensis
Biopsia	B246	38	M	Brucella melitensis
Estirpe	B247	65	M	Brucella mellitensis
Sangue	B248	0	F	Negativo

Estirpe	B249	60	F	Brucella mellitensis
Liquor/LCR	B250	56	M	Negativo
Liquor/LCR	B251	50	M	Negativo
Liquor/LCR	B252	63	F	Negativo
Liquor/LCR	B253	71	M	Negativo
Sangue	B254	43	F	Negativo
Liquor/LCR	B255	73	M	Negativo
Liquor/LCR	B256	69	M	Negativo
Biopsia	B257	50	M	Negativo
Medula óssea	B258	50	M	Negativo
Sangue	B259	9	M	Negativo
